# Mitochondrial and cardiovascular responses to aerobic exercise training in supine and upright positions in healthy young adults: a randomized parallel arm trial

**DOI:** 10.1515/teb-2025-0002

**Published:** 2025-03-26

**Authors:** Nicholas Preobrazenski, Stuart P.S. Mladen, Ejaz Causer, Eveline Menezes, Hashim Islam, Patrick J. Drouin, Michael E. Tschakovsky, Brendon J. Gurd

**Affiliations:** School of Kinesiology and Health Studies, 4257 Queen’s University, Kingston, ON, Canada; Faculty of Medicine, University of Ottawa, Ottawa, ON, Canada; School of Health and Exercise Sciences, University of British Columbia – Okanagan, Kelowna, BC, Canada

**Keywords:** *PGC-1α*, supine exercise, exercise performance, aerobic training, cardiovascular response

## Abstract

**Objectives:**

Aerobic exercise training can increase skeletal muscle mitochondrial content. Supine exercise training with legs above the heart potentially augments these increases. However, the impact of supine exercise training on mitochondrial biogenesis and cardiovascular adaptations remains unclear.

**Methods:**

In this single-centred, randomized, parallel arm trial, 19 recreationally active individuals underwent seven sessions of either supine with legs up (SUP; n=9, 6 females) or upright with legs down (UP; n=10, 7 females) aerobic training on a recumbent bike at 71 ± 7 % and 71 ± 2 % of peak work rate (WR_peak_), respectively. The study aimed to test the effects of training with decreased muscle oxygenation on indices of muscle mitochondrial remodelling. Secondary outcomes included exercise performance, muscle oxygenation, and cardiovascular responses.

**Results:**

Secondary outcomes revealed significant interaction effects for time to fatigue (TTF) and WR_peak_ in the SUP group during supine testing, suggesting enhanced exercise tolerance and performance. No between group interaction effects were observed for upright testing. No clear effects on mitochondrial biogenesis were observed based on expression of mitochondrial protein subunits and transcriptional regulators. Acutely, HR_peak_ was lower during the SUP Test compared to the UP Test. No central cardiovascular adaptations were observed following training.

**Conclusions:**

Our exploratory analyses showed that supine aerobic training more effectively improves supine exercise tolerance and performance compared with upright training, despite no differences in measured proteins related to mitochondrial biogenesis. Further research is needed to elucidate the mechanisms underlying these postural-specific training effects.

**Registration:**

clinicaltrials.gov: NCT04151095.

## Introduction

Aerobic exercise training increases skeletal muscle mitochondrial content [1]. Signals generated by muscle contraction activate peroxisome proliferator-activated receptor gamma coactivator-1 alpha (PGC-1α), which coordinates the expression of mitochondrial and nuclear genomes [2], 3]. PGC-1α activation (based on changes in its mRNA [4]) is intensity-dependent following submaximal exercise intensities [5], 6], and bursts of *PGC-1α* mRNA following aerobic exercise precede increased mitochondrial proteins in human skeletal muscle [7]. Interestingly, blood flow restricted (BFR) aerobic exercise increases *PGC-1α* mRNA more than work rate-matched aerobic exercise [8], [9], [10]. Although augmented *PGC-1α* mRNA indicates greater activation of mitochondrial biosynthetic pathways, it is unknown whether larger bursts of *PGC-1α* mRNA during BFR aerobic training lead to larger increases in mitochondrial content [11], 12].

**Figure 1:**
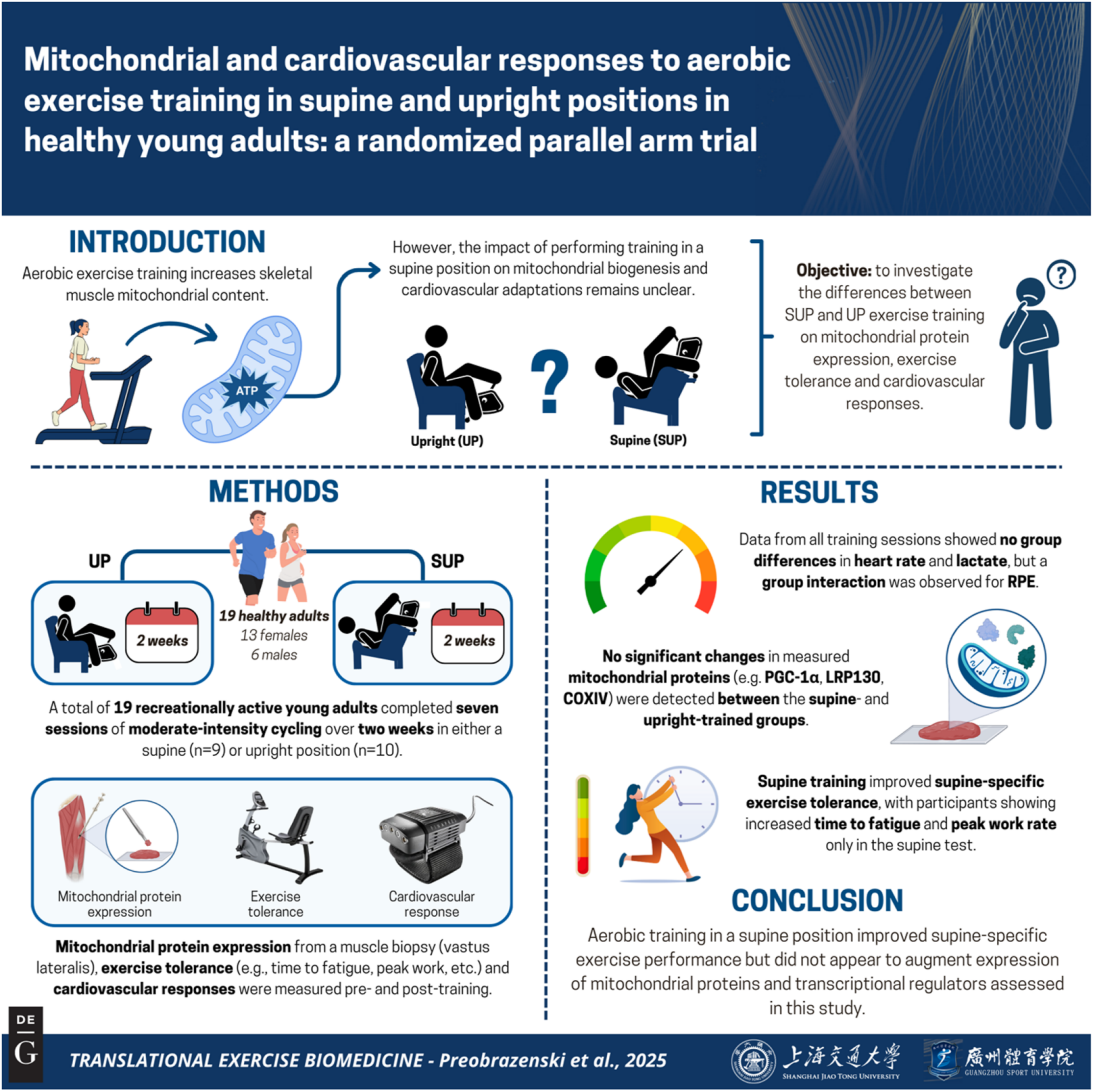
Graphical representation of this study. Key points: (1) this research tested whether cycling with legs above the heart enhances mitochondrial biogenesis. It attempted to bridge gaps between standard aerobic training and more accessible ‘blood flow restriction’ models. (2) Nineteen healthy, recreationally active adults completed seven sessions of moderate intensity cycling in either supine or upright positions. We tested the hypothesis that supine training would augment citrate synthase compared with upright training. (3) Supine training enhanced exercise tolerance in the supine position but did not alter measured mitochondrial protein expression, suggesting posture-specific improvements without detected changes in mitochondrial proteins. These results may guide new approaches to posture-based aerobic training. Figure created with BioRender.

Studies comparing BFR with regular aerobic training report larger increases in skeletal muscle citrate synthase maximal activity, a biomarker of mitochondrial content [13], following BFR [14], [15], [16]. However, results from two of these studies [14], 15] should be interpreted cautiously because they lack pre-training data. The aforementioned studies [14], [15], [16] also used expensive and difficult-to-acquire lower body positive pressure chambers. We therefore built an inexpensive and feasible model that reduces muscle oxygenation by placing the exerciser’s legs above the heart while cycling in a supine position (SUP) [17]. Acute aerobic exercise using our supine model increased *PGC-1α* mRNA (and presumably PGC-1α activation) more than work-matched upright aerobic exercise [17], which is consistent with studies using BFR [8], 10]. Therefore, the intended purpose of this study was to test the hypothesis that aerobic training with legs above the heart (SUP) increases citrate synthase activity more than work rate-matched, aerobic training with legs below the heart (UP). Determining whether supine exercise can augment exercise adaptations without increasing workload may have practical implications for populations where high-load exercise is contraindicated. Secondary outcomes were time to fatigue (TTF), peak work rate (WR_Peak_), peak heart rate (HR_Peak_), central cardiovascular responses, and expression of mitochondrial protein subunits and transcriptional regulators. Figure 1 presents a summary of this article.

## Materials and methods

### Participants

**Figure 2:**
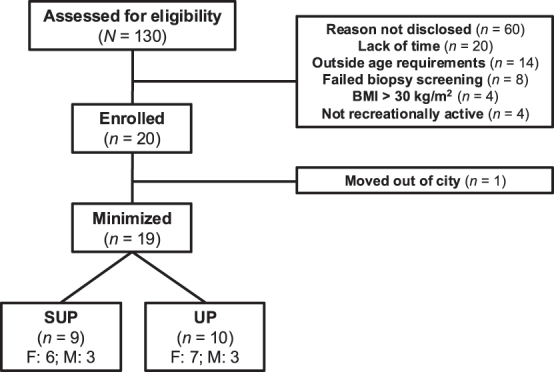
Flow chart depicting recruitment, minimization, drop-outs, and completion. *Note:* SUP, aerobic training in a supine position with legs above the heart; UP, aerobic training with legs below the heart; BMI, body mass index.

Twenty healthy young females (n=13) and males (n=7) enrolled in the current study (Figure 2). Inclusion criteria were: 18–30 years of age, recreationally active (self-reported, less than 3 h of structured physical activity per week), no previous cycling training, no concurrent involvement in another exercise training program, and body mass index <30 kg/m^2^. Exclusion criteria were: presence of cardiovascular or metabolic disease, taking oral medication, and current smoker. Physical activity levels and readiness were assessed using a 7-day Physical Activity Recall Questionnaire and the Physical Activity Readiness Questionnaire for Everyone, respectively. All participants attended a preliminary screening session where they were provided a verbal and written explanation of the experimental protocol and its associated risks before providing informed consent and participating in the study. This study was approved by The Health Sciences Human Research Ethics Board at Queen’s University, Kingston, Ontario, Canada (ref. no: 6018724), and it conformed to the *Declaration of Helsinki*.

### Experimental design

A single-centred, randomized, parallel arm study design was used to examine the fold change in citrate synthase maximal activity (primary outcome) following aerobic training on a recumbent bike in a supine position with legs above the heart (SUP) or upright position with legs below the heart (UP) (clinicaltrials.gov: NCT04151095). Blood flow was not directly measured, and the confirmed difference between groups was body positioning during exercise training. Thus, the terms ‘BFR’ and ‘CTL’ appearing on the trial registration have been replaced with SUP and UP, respectively.

**Figure 3:**
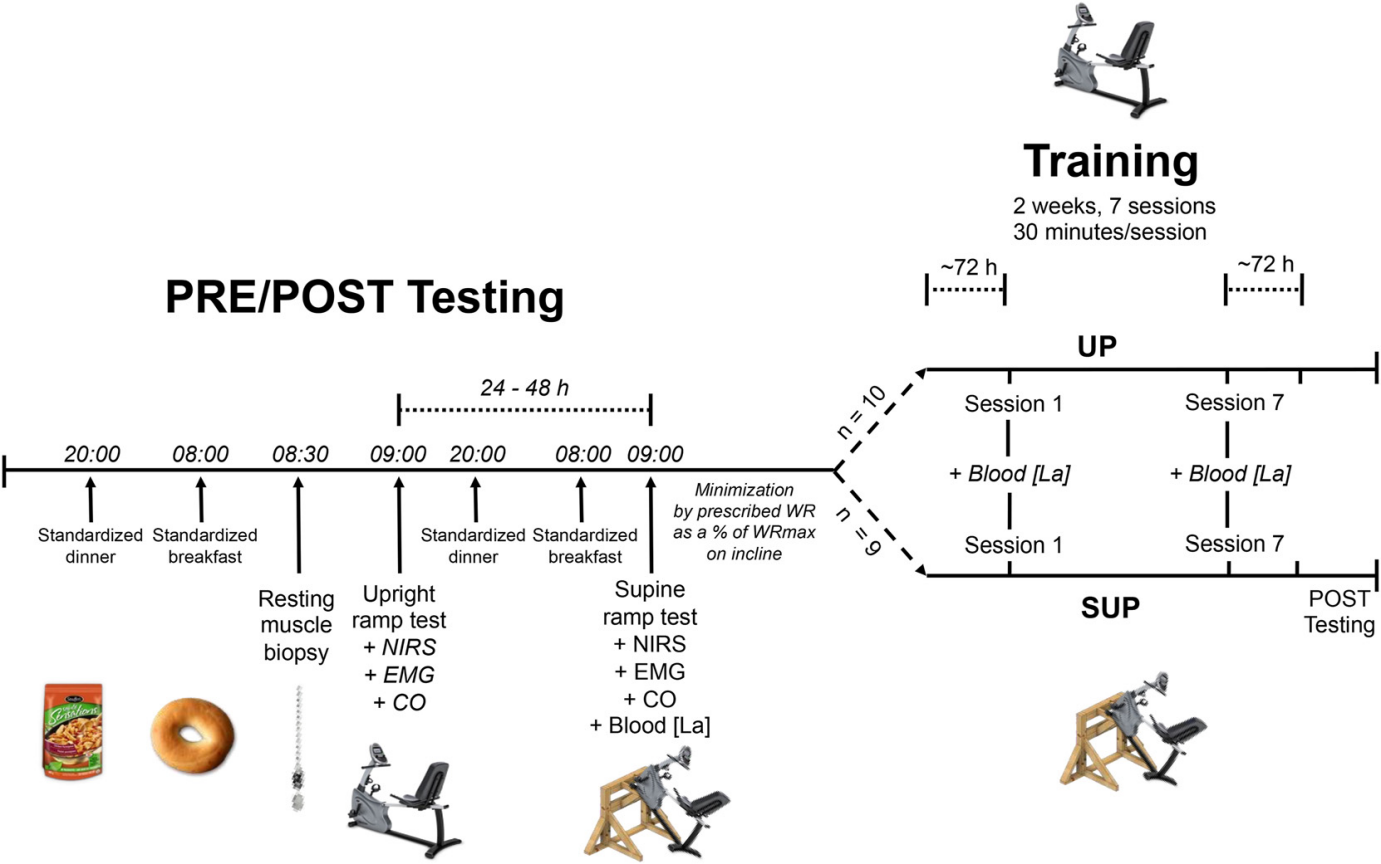
Experimental timeline. Participants underwent identical testing protocols at PRE and POST. Note: CO, cardiac output; EMG, electromyography; [La], blood lactate concentration; NIRS, near-infrared spectroscopy; PRE, baseline; POST, post-intervention; SUP, aerobic training in a supine position with legs above the heart; UP, aerobic training with legs below the heart; WR, work rate.

Data collection took place between May 6, 2020 and January 30, 2021 in the Queen’s Muscle Physiology Lab in Kingston, Ontario. The first participant was randomly allocated to SUP or UP aerobic training using a random number generator (MS Excel 2016; Redmond, Wash., USA). Subsequent participants were allocated in pairs to SUP (n=10) or UP (n=10) aerobic training in a manner that minimized [18] the imbalance of prescribed work rate (WR) as a % of WR_peak_ achieved in the ramp test performed in the SUP position (see [17] for images of exercise equipment positioning). For example, if participant A had a higher WR as a % of WRpeak than participant B, and the SUP group had a lower average WR % than the UP group, participant A would be allocated to SUP to minimize the imbalance between groups. All participants completed two ramp tests (see *PRE/POST Testing*) in the week preceding (PRE) the first training session and the week following the last training session (POST). One ramp test was performed with legs below (UP Test), and the second test was performed with legs above the heart (SUP Test). Allocation was not revealed until immediately before the first training session. Figure 3 depicts the experimental timeline.

All exercise was performed on the same electronically braked Vision Fitness R10 recumbent bike (Cottage Grove, Wis., USA). The same two investigators (N.P and E.C) conducted physiological testing and provided the one-on-one, supervised exercise training. Verbal encouragement was provided to all participants at least every 2 min throughout each testing and training session. Participants reported they were not taking any nutritional supplements at the time of the study. Participants were asked to refrain from exercising for 24 h before, and from alcohol and caffeine for 12 h before, all testing and training sessions. Participants were also asked to maintain habitual physical activity levels throughout the trial. PRE and POST testing along with exercise training occurred at the same time of day (±1 h) to reduce potential variability related to timing. The study implemented the same rigorous methodological considerations outlined in the Supplementary Document S11 of [17]. These considerations included computer-generated randomization, allocation concealment, outcome assessor blinding, and trial registration to mitigate risks of selection, observer, and outcome reporting biases, respectively.

### PRE/POST testing

Participants reported to the Queen’s Muscle Physiology Lab four times in total for testing. They completed two ramp tests at PRE and two at POST with each pair of visits separated by at least 48 h. The present study used the PRE/POST testing protocols detailed in [17], however gas exchange was not measured in this study. Briefly, during the first testing visit, participants completed a ramp test on an electronically braked Vision Fitness R10 recumbent bike placed on flat ground (UP Test). The ramp test consisted of a 5-min warm-up at 70–75 RPM at ∼58 W, followed by ∼15 W increases every 3 min until volitional failure (i.e. when the participant could no longer maintain a cadence of 70–75 RPM). This ramp test characterized physiological responses to normal intra-exercise muscle oxygenation. During the second testing visit, participants completed a ramp test with the recumbent bike placed on a 45° incline (SUP Test). Fingertip capillary blood (∼20 μL) was collected at rest and within the last 30 s of each successive 3-min stage of the SUP Test for measurement of lactate concentration, using a Lactate Scout+ (EKF Diagnostics, Madgeberg, Germany), which is a device with acceptable accuracy and reliability [19]. The SUP Test familiarized participants with the SUP exercise stimulus (i.e. reduced intra-exercise muscle oxygenation) and determined the onset of blood lactate accumulation (OBLA) (blood lactate concentration of at least 4.0 mmol/L) [20]. OBLA during the PRE SUP Test was used to prescribe exercise intensity during aerobic training (see *Training protocol*). Both ramp tests provided a time-to-fatigue (TTF) value which reflected duration on a stopwatch from test onset to volitional failure. Heart rate (HR), WR, revolutions per minute (RPM), rating of perceived exertion (RPE), and leg-specific ratings of perceived pain (RPP-leg) were recorded in the last 30 s of every 3-min stage during the ramp tests using a previously described protocol [17]. If a ramp test ended prior to the last 30 s of each stage, values immediately following volitional exhaustion were recorded.

### Training protocol

Training consisted of seven individual and fully supervised, 30-min training sessions over 2 weeks. Each training day included only one session. Participants in both SUP and UP training groups exercised at the WR (i.e. level 1–16 at 70–75 RPM) immediately preceding their OBLA during the SUP Test. Heart rate (HR) was monitored continuously throughout each training session using Polar HR monitors (Polar Team2 Pro, Kempele, Finland). HR, WR, RPM, RPE, and leg-specific ratings of perceived pain were recorded in the last 30 s of each 5-min period throughout training sessions. Fingertip blood draws were also taken at the 10th and 30th minutes into exercise during the first and last training sessions using the Lactate Scout+.

### Data collection

#### Cardiovascular responses

During all PRE/POST tests, a finger photoplethysmograph (Finometer MIDI; Finapres Medical Systems, Enschede, The Netherlands), placed on the participants left middle finger and kept at heart level, was used to obtain the following beat-by-beat cardiovascular measurements: cardiac output (CO), stroke volume (SV), heart rate (HR), mean arterial pressure (MAP), systolic blood pressure (SBP), diastolic blood pressure (DBP) and total peripheral conductance (TPC). Thirty second time bins were calculated for all cardiovascular variables. The final time bin of each stage was averaged across participants, and the highest average value was selected as peak measurement of each variable.

#### Muscle oygenation

Vastus lateralis muscle oxygenation – change in oxygenated (oxy [haem]) and deoxygenated (deoxy [haem]) hemoglobin – was recorded via near-infrared spectroscopy (NIRS; Oxymon MK III, Artinis Medical Systems, Elst, Netherlands), at a location two-thirds down the line from the anterior superior iliac spine to the lateral patella. NIRS data was collected using methods described elsewhere [17], 21].

#### Perception of effort and leg pain

The Borg CR10 scale was used to measure both perception of effort and leg pain as described elsewhere [17]. Perceived effort and leg pain was recorded during the final 30 s of each stage for each participant. If a ramp test ended prior to the last 30 s of each stage, values immediately preceding volitional exhaustion were recorded.

#### Muscle biopsy

Participants reported to the lab in the morning after (∼12 h) an overnight fast. Prior to the ramp tests at PRE and POST, participants were fed a standardized dinner (Yakitori Chicken with Japanese-Style Fried Rice (360 kcal; 56 g carbohydrates (CHO), 8 g fat, 15 g protein) and 500 mL of 1 % chocolate milk (340 kcal; 28 g CHO, 5 g fat, 18 g protein)) and a standardized breakfast (12 grain bagel (230 kcal; 5 g fat, 38 g CHO, 10 g protein) with 1.5 oz (42.5 g) of cream cheese (150 kcal; 15 g fat, 1 g CHO, 3 g protein) and 300 mL of orange juice (130 kcal; 32 g CHO, 0 g fat, 1 g protein)). Thirty minutes after finishing breakfast, a resting muscle biopsy was taken from the lateral portion of the left *vastus lateralis*. The ramp test began ∼30 min after the biopsy on the first testing visit. Exercise began ∼60 min after finishing breakfast on the second testing visit to match timing of the first testing visit.

Biopsies were obtained using a Bergström needle with manual suction while the participant lay in a supine position (described in [22]). PRE and POST biopsies were obtained ∼ two cm distal to each other on the left leg. ﻿A portion of each muscle biopsy was embedded in Tissue-Tek^®^ O.C.T. Compound (#4583, Sakura Finetek, St, Torrance, CA) and frozen in liquid nitrogen-cooled isopentane for potential histochemical analysis. However, planned histochemical analyses were not attempted. Remaining muscle tissue was immediately snap frozen in liquid nitrogen and stored at −80 °C until Western blotting analysis.

#### Western blot analysis

Western blotting was performed as previously described [23] on whole muscle homogenates using commercially available antibodies against total OXPHOS (1:1000 dilution, Abcam, ab110411), LRP130 (1:1000 dilution, Abcam, ab97505), COXIV (1:1000 dilution, Abcam, ab14744), NRF2 (1:1000 dilution, Cell Signalling, DIZ9C XP), and PGC-1﻿α (1:1000, EMD Millipore, AB3242). Although planned, transcription factor A, mitochondrial (TFAM) was never blotted. Blots are presented in the Supplementary Material. Equivalent total protein was loaded for each sample. Amido black staining was used for total protein normalization to confirm equal loading across lanes, rather than using a single housekeeping protein as an internal control. No significant differences were observed between conditions or time points (all p>0.05). All protein data are expressed relative to total protein loaded.

### Statistical analysis

Descriptive statistics were used to summarize baseline participant characteristics. Paired *t*-tests were performed to assess differences between upright and supine ramp tests at PRE. Due to missing data from measurement errors (assumed to be missing at random), mixed effects models were employed to analyze changes in cardiovascular variables over time between the upright-trained and the supine-trained groups. Two-way repeated measures ANOVAs were conducted to assess between-group changes in RPM, HR, RPE, RPP-leg, and lactate during the training sessions as these variables had complete data. Similarly, two-way repeated measures ANOVAs were performed to evaluate changes over time in WR, TTF, and whole-muscle protein content between training groups (complete data). Separate ANOVAs were used for the SUP and the UP ramp tests.

Consistent with CONSORT recommendations [24], we calculated and reported effect sizes using partial eta-squared (η^2^_*p*_) and Cohen’s *d* to contextualize physiological differences and to facilitate sample size calculations in future work. Significant interaction or main effects within each ANOVA were subsequently analyzed using Bonferroni *post hoc* tests to control for Type I error inflation among multiple comparisons within each ANOVA. Statistical significance was set at p<0.05 given hypotheses for secondary outcomes were not specified *a-priori*. A higher experiment-wise error rate was accepted given the exploratory, hypothesis-generating nature of the current paper [25]. All data are presented as means ± standard deviation.

An *a priori* sample size calculation was calculated for the primary outcome: within-between interaction for fold change in CS activity. G*Power 3.1 revealed that a sample size of 18 was required to detect a statistically significant (p<0.05) moderate effect size (*f*=0.25) between SUP and UP with 80 % power (correlation among repeated measures=0.75 (conservative estimate based on data from [26]), ⍺ error probability=0.05, 1 – β error probability 0.8).

Statistical analyses were conducted using GraphPad Prism version 9.4.1 (GraphPad Software, San Diego, Calif., USA) and JASP version 0.18.3 (JASP Team, Amsterdam, NL). All tests were two-tailed.

## Results

Participant recruitment and enrolment are described in Figure 2. Nineteen participants provided muscle biopsies at PRE and POST, and western blotting was conducted in 19 participants. Citrate synthase analysis could not be completed due to a lack of funds and tissue to repeat the initial citrate synthase analyses which yielded unusable data. Sample sizes for cardiovascular outcomes were less than 19 (n=12–14) due to measurement error causing data loss, and these missing data are reported in the tables. Table 1 presents participant characteristics obtained during PRE.

**Table 1:** Pre-training participant characteristics (total: n=19; UP: n=10; SUP: n=9).

	Total	UP-trained	SUP-trained	Cohen’s *d*_*s*_ (p-Value)
Sex	F: 13; M: 6	F: 7; M: 3	F: 6; M: 3	–
Age, years	20.9 ± 2.3	20.8 ± 2.3	21.1 ± 2.4	−0.13 (0.78)
Height, cm	167.9 ± 11.0	169.0 ± 12.1	166.8 ± 10.1	0.20 (0.67)
Weight, kg	66.2 ± 11.9	65.8 ± 13.9	66.5 ± 10.0	−0.06 (0.90)

All comparisons were UP minus SUP with 17° of freedom (two-tailed, independent groups). Values are means ± standard deviation. SUP, aerobic exercise training in a supine position with legs above the heart; UP, aerobic exercise training in an upright position with legs above the heart.

### Exercise training characteristics

Nineteen participants completed all seven exercise sessions. The SUP and UP groups were prescribed exercise at 71 ± 7 % and 71 ± 10 % of WR_peak_ attained from the PRE supine ramp test, respectively. There were significant effects of time (p<0.001; η^2^_*p*_=0.56) and time × group interaction (p<0.001; η^2^_*p*_=0.57) for the prescribed WR as a % of WR_peak_. The SUP group was exercising at 60.1 ± 4.8 % of WR_peak_ by session seven (p<0.001; *d*_*z*_= −1.32). Mauchly’s test indicated a violation of sphericity for average session RPM (p<0.05). The Greenhouse-Geisser correction was applied, and there were no effects of time (*p*_*G−G*_=0.48; η^2^_*p*_=0.047), group (p=0.34; η^2^_*p*_=0.053), or time × group interaction (*p*_*G−G*_=0.066; η^2^_*p*_=0.13) for training session RPM.

**Figure 4:**
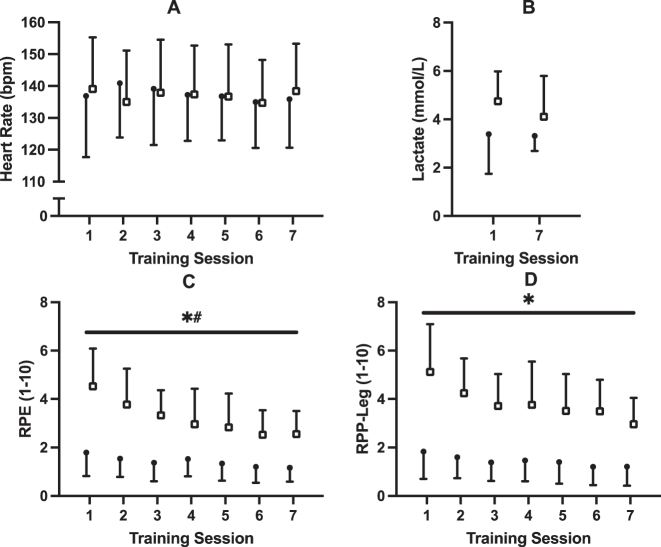
Training data for (A) heart rate, (B) blood lactate, (C) rating of perceived exertion, and (D) rating of perceived pain in legs. Participants underwent seven, 30 min sessions over 2 weeks with either upright (UP; n=10; closed circles) or supine (SUP; n=9; open squares) aerobic training on a recumbent bike. * Significant (p<0.001) main effect of group ^#^ significant time × group interaction (p<0.05).

HR, RPE, RPP-leg, and blood lactate data for all training sessions are presented in Figure 4. No effects of time (p=0.74; η^2^_*p*_=0.033), group (p=0.96; η^2^_*p*_<0.001), or time × group interaction (p=0.59; η^2^_*p*_=0.044) were observed for HR (see Figure 4A). No effects of time (p=0.23; η^2^_*p*_=0.082), group (p=0.068; η^2^_*p*_=0.18), or time × group interaction (p=0.33; η^2^_*p*_=0.055) were observed for blood lactate (see Figure 4B). Mauchly’s test indicated a violation of sphericity for RPE and RPP-leg (p<0.05). The Greenhouse-Geisser correction was applied, and significant effects of time (*p*_*G−G*_<0.001; η^2^_*p*_=0.39), group (p<0.001; η^2^_*p*_=0.54) and time × group interaction (*p*_*G−G*_=0.032; η^2^_*p*_=0.172) remained for RPE (See Figure 4C). Significant effects of time (*p*_*G−G*_<0.001; η^2^_*p*_=0.273) and group (p<0.001; η^2^_*p*_=0.62) were observed for RPP-leg, but no time × group interaction (*p*_*G−G*_=0.17; η^2^_*p*_=0.097) was observed (See Figure 4D).

### Ramp test performance

**Figure 5:**
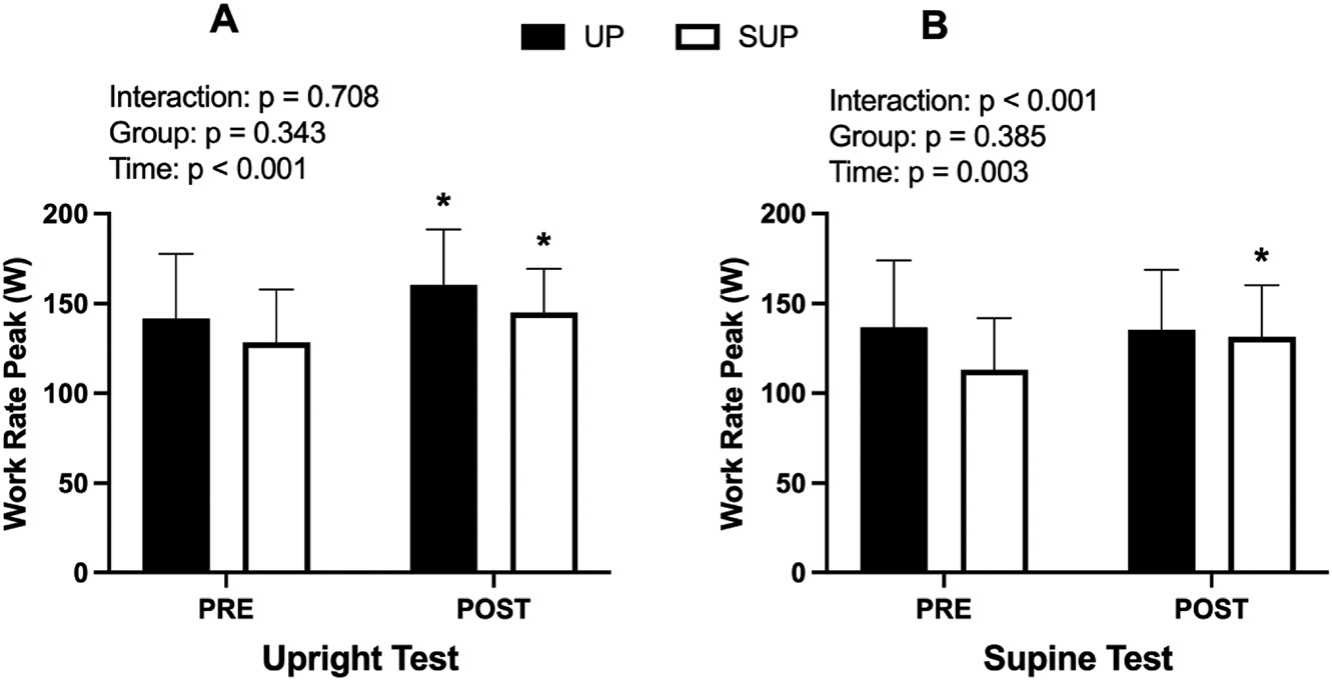
Work rate peak (W) data for the (**A**) upright and (**B**) supine incremental ramp test before (PRE) and after (POST) training. Data from both the upright- (UP; n=10; closed bars) and supine-trained (SUP; n=9; open bars) groups are presented. *Significant within-group difference from PRE.

WR_peak_ data for the PRE and POST upright and supine ramp tests are presented in Figure 5 and Table 2. A significant effect of time (p<0.001; η^2^_*p*_=0.73) but no effect of group (p=0.53; η^2^_*p*_=0.024) or group × time interaction (p=0.98; η^2^_*p*_<0.001) was observed for WR_peak_ during the upright test (See Figure 5A). *Post hoc* tests revealed significant increases in WR_peak_ from PRE to POST on the upright test for both UP (p<0.001; *d*_z_=0.54) and SUP (p<0.001; *d*_z_=0.54) groups. For the supine test, a significant effect of time (p<0.001; η^2^_*p*_=0.51) and group × time interaction (p<0.001; η^2^_*p*_=0.57) were observed. There was no effect of group (p=0.61; η^2^_*p*_=0.016) for the supine test (See Figure 5B). *Post hoc* tests revealed significant increases in WR_peak_ for the supine test in the SUP (p<0.001; *d*_z_=0.61) but not the UP group (p=0.71; *d*_z_=0.034).

**Table 2:** Pre- and post-training ramp test performance (total: n=19; UP: n=10; SUP: n=9).

	PRE	POST	UP-trained		SUP-trained		Time	Group	T × G
			PRE	POST	PRE	POST			
*Upright ramp test*									
TTF, s	1242 ± 361^a^	1433 ± 370	1290 ± 411	1501 ± 430^b^	1189 ± 311	1358 ± 297^b^	**<0.001**	0.48	0.41
Max level (1–16)	5.9 ± 2.1^a^	7.0 ± 2.0	6.2 ± 2.4	7.3 ± 2.3^b^	5.6 ± 1.8	6.7 ± 1.6^b^	**<0.001**	0.50	0.97
WR_peak_, W	134 ± 32^a^	151 ± 29	138 ± 36	155 ± 34^b^	129 ± 28	146 ± 23^b^	**<0.001**	0.53	0.98

*Supine ramp test*									
TTF, s	1105 ± 405	1223 ± 385	1198 ± 451	1202 ± 416	1001 ± 341	1247 ± 372^b^	**<0.001**	0.68	**<0.001**
Max level (1–16)	5.2 ± 2.3	5.8 ± 2.0	5.8 ± 2.6	5.7 ± 2.3	4.6 ± 1.7	5.9 ± 1.8^b^	**<0.001**	0.60	**<0.001**
Prescribed level (1–16)	2.8 ± 1.3		3.1 ± 1.4		2.4 ± 1.1				
WR at prescribed level, W	86 ± 20		91 ± 21		80 ± 18				
WR at level of OBLA, W	100 ± 25	101 ± 25	105 ± 25	107 ± 27	95 ± 25	94 ± 22	0.85	0.30	0.83
WR_peak_, W	123 ± 34	132 ± 31	132 ± 38	131 ± 35	114 ± 27	134 ± 28^b^	**<0.001**	0.61	**<0.001**
Prescribed WR as % of WR_peak_, %	70.9 ± 8.7	65.7 ± 9.3	70.7 ± 10.3	70.8 ± 9.6	71.1 ± 7.0	60.1 ± 4.8^b^	**<0.001**	0.18	**<0.001**

^a^Significant difference between the Upright and Supine tests at PRE. ^b^Significant within-group difference between PRE and POST. Values are means ± standard deviation. Bolded values represent p-values <0.05. OBLA, onset of blood lactate accumulation; POST, post training; PRE, pre-training; SUP, supine training; T × G, time by group interaction effect; TTF, time to fatigue; UP, upright training; WR, work rate.

Analyses run on ramp test time to fatigue (seconds) revealed identical statistical outputs as the WR_peak_ data presented above (Table 2). For simplicity we have only presented WR_peak_ data here and in Figure 5.

### Western blot

**Figure 6:**
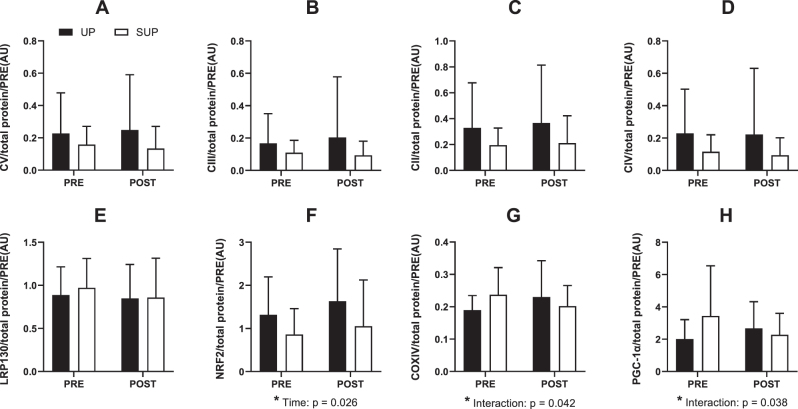
Protein expression from whole muscle homogenate of human *vastus lateralis* before (PRE) and after (POST) 2 weeks of upright (black boxes) or supine (white boxes) aerobic training (n=19). (**A**) Complex V, (**B**) Complex III, (**C**) Complex II, (**D**) Complex IV, (**E**) leucine-rich protein 130, (**F**) nuclear factor erythroid 2-related factor 2, (**G**) Cytochrome *c* oxidase subunit IV, and (**H**) ﻿peroxisome proliferator-activated receptor gamma coactivator-1 alpha. All protein data are expressed relative to total protein. Data are presented as means ± standard deviation. Main effects and interactions (two-way repeated measures ANOVA) are reported. No post-hoc differences were observed for significant interaction effects.

Results for all western blot analysis are presented in Figure 6. There were no significant differences in baseline protein expression between groups for any of the protein examined (all p<0.05). A significant effect of time was observed for NRF2 (p=0.026; η^2^_*p*_=0.26). A significant group × time interaction effect was observed for PGC-1⍺ (p=0.038; η^2^_*p*_=0.23). Bonferroni’s post-hoc tests revealed no significant PRE-POST changes for the UP-trained (p=0.51) or SUP-trained group (p=0.13). A significant group × time interaction effect was observed for COXIV (p=0.042; η^2^_*p*_=0.22). Bonferroni’s post-hoc tests revealed no significant PRE-POST changes for the UP-trained (p=0.21) or SUP-trained group (p=0.35). No other significant main or interaction effects were observed. Supplemental Figure 1 presents a representative blot.

### Hemodynamic responses

Supplemental Table 1 presents peak hemodynamic responses for both ramp tests between the UP-trained and SUP-trained groups. Acutely, HR_peak_ was lower during the supine compared to the upright ramp test (p=0.02*; d*_*z*_=0.97). Significant effects of time were observed for oxy [haem] in the upright (p=0.04) and supine (p<0.01) ramp tests. There were significant group × time interaction effects for the supine ramp test in CO (p=0.04) and oxy [haem] (p=0.03). Post-hoc tests revealed significant PRE-POST differences in oxy [haem] for the UP-trained group (p=0.004) but not the SUP-trained group (p=0.99). There were no significant post-hoc comparisons in CO for the UP-trained (p=0.61) or SUP-trained group (p=0.10). No other significant effects were observed for peak hemodynamic responses (see Supplemental Table 1 for p-values).

## Discussion

In this randomized controlled trial, 19 participants underwent seven, 30-min sessions of moderate intensity aerobic training in either a supine position with legs above the heart (SUP) or upright position with legs below the heart (UP). Although we previously observed augmented AMPK and PGC-1α activation during bouts of supine cycling [17], this augmentation did not translate into changes in the mitochondrial proteins we assessed following seven training sessions. In fact, participants in SUP had smaller changes in PGC-1α and COXIV compared with those in UP. Nonetheless, secondary outcomes presented in this paper can provide additional understanding of the physiological adaptations to aerobic exercise with legs above the heart. For instance, SUP-Training may enhance exercise tolerance and performance more than UP-Training during the SUP test. While the postural effects observed in our model contribute to understanding of exercise posture on cardiovascular adaptations, they also support a need for further research to elucidate the specific mechanisms by which training with legs above the heart influences cardiovascular function and exercise performance.

### Reducing risk of bias with experimental best practices

This paper can serve as an example for why pre-registration should be maintained in experimental physiology (see opposing viewpoint in [27]). Without pre-registration, we could have omitted reference to citrate synthase activity and instead centered this paper around the significant changes in work rate peak to maximize chances of publication (i.e. selective reporting with Hypothesizing After the Results are Known). This is why we, and many others (e.g. [28], 29]), advocate for pre-registration.

While unforeseen circumstances disrupted our intended plan, assessing secondary outcomes understandably requires thorough justification and tempers our conclusions. Implementing measures against biases, including reporting bias, not only enhances the credibility of science [30] but can also conserve resources [31] and minimize potential harm to participants [32]. To reduce risk of selection and detection biases, we concealed allocation and blinded assessors. We did not apply multiplicity adjustments to correct for multiple comparisons at the main ANOVA level as our aim was exploratory hypothesis generation rather than confirmatory testing. This decision preserves the conventional α=0.05 for each individual test but increases the risk of Type 1 error across all comparisons. Without correcting for multiple comparisons, the risk of falsely declaring at least one significant result increases from 5 % for a single test to ∼81 % [i.e. 1−(1−0.05)^32^] across all 32 2 × 2 ANOVAs conducted. Any significant findings should be interpreted cautiously as they may represent a false positive. The exploratory nature of our underpowered secondary analyses clearly precludes broad or definitive claims, and further pre-registered confirmatory studies with larger sample sizes and targeted primary outcomes are needed to test hypotheses arising from our results.

### Pre-training peak WR and hemodynamic responses during SUP and UP ramp tests

Our observations of lower WR_peak_ during SUP Test cycling compared with UP cycling (Table 2) align with previous studies showing lower VO_2peak_ and WR_peak_ during SUP [33], 34]. Although leg blood flow was not measured, these differences in performance likely stem from a reduction in exercising perfusion pressure in the SUP condition caused by decreased gravitational assistance to muscle perfusion. Indeed, a previous study from our group shows our SUP model lowers muscle oxygenation [17]. With impaired oxygen delivery, redox and phosphorylation potentials must be adjusted to maintain ATP production. However, this leads to increased accumulation of ADP, Pi, and H^+^ [35], ultimately accelerating the rate of muscle fatigue development [36] and potentially contributing to the lower WR_peak_ during SUP cycling. The decrease in HR_peak_ during SUP cycling, without significant changes in CO_peak_ or SV_peak_, underscores the underpowered nature of our study to assess cardiovascular adaptations. A decreased HR should result in either a decreased cardiac output or a maintained cardiac output with an increased SV. Although statistically insignificant, SV_peak_ was 11.5 mL/min higher during the supine test than the upright test. Indeed, several studies have observed increased SV in response to transitions from upright to supine or head-down postures due to changes in central venous pressure and preload [37], [38], [39].

The equivocal effects of posture on CO during exercise highlight the complexity of cardiovascular adaptations to SUP. While our results align with studies reporting no significant difference in CO between SUP and UP cycling [37], 38], other research has observed increased CO during SUP cycling [40]. This discrepancy may arise from differences in exercise protocols, specific postural positions, and individual physiological responses to SUP. Furthermore, while we observed a reduction in both MAP and SBP during SUP cycling compared with UP (Supplemental Table 2), the cause of this effect remains uncertain. This uncertainty persists despite non-significant changes in key determinants of blood pressure, including CO and TPC.

### Influence of posture on training adaptations

The training adaptations observed in our study highlight the posture-specific effects of supine training. Only the supine-trained group increased WR_peak_ during the supine test, whereas both groups increased WR_peak_ during the upright test. The results of this study align with previous research [37], 39] and suggest that adaptations from supine training generalize to upright posture, while adaptations gained from upright training do not generalize to supine posture. Although our study offers limited data to fully explain the mechanisms that underlie these adaptations, the significant reduction in RPE observed in the supine-trained group during the supine test suggests that supine positioning may improve tolerance to this form of cycling, thereby leading to increased TTF and WR_peak_.

Despite posture-specific training adaptations to WR_peak_, the lack of significant central cardiovascular adaptations following training in SUP contrasts with other research [37]. However, the duration of our training intervention was comparably short (2 vs. 4 weeks), and thus it is unclear whether central adaptations would arise following a longer training intervention. Alternatively, this discrepancy could be attributed to the differences in intensity, statistical power, methodological techniques, or differences in perfusion pressure reductions. While vascular O_2_ content can also augment convective O_2_ transport, findings elsewhere [39] show no significant changes in blood or red blood cell volume after training in a supine posture. This supports the notion that improvements in aerobic capacity following supine exercise training may not necessarily be mediated by increases in O_2_ content or significant alterations in blood volume. The absence of central cardiovascular adaptations in our study suggest that peripherally based mechanisms may underpin the observed posture-specific training adaptations. For instance, improvements in oxygen delivery following exercise training are often attributed to O_2_ diffusional capacity enhancements in muscle microcirculation, such as increased capillary blood flow [37].

Our model diverges from the traditional supine or head-down tilt postures by not creating a true head-down tilt. Instead, our model elevated the legs above heart level without fully inclining the midsection. This unique posture might not elicit the same physiological responses as a complete head-down tilt or supine position, possibly due to differences in hydrostatic pressure gradients and venous return. Consequently, the adaptations in cardiovascular function and muscle perfusion with our model may differ from those observed in traditional postures. By exploring these differences, our study broadens the understanding of how posture impacts cardiovascular adaptations following aerobic exercise training.

### Limitations and future directions

Due to unforeseen circumstances, we were unable to measure the primary outcome for which our study was powered. While we pre-specified several outcomes appearing in this paper (e.g. NIRS, EMG, HR), we did not pre-specify others (nor their hypothesized changes) such as time to fatigue, TPC, CO, etc. We transparently disclose this information to mitigate risk of reporting bias, and we do not make confirmatory statements regarding our outcomes. The prevalence of risk of bias in exercise science [41] highlights a need for future research needs to directly assess mitochondrial content and function following supine aerobic training using experimental best practices.

In the present study, exercise training intensity in both groups was prescribed at the WR immediately preceding the OBLA during the pre-training SUP WR_peak_ test, and participants were allocated to minimize differences in WR as a % of SUP WR_peak_. We intentionally matched between-group workload since one of the applications of supine or BFR exercise is the potential to augment adaptations without increasing workload, which is beneficial for individuals for whom high-load exercise is contraindicated. However, it should be acknowledged that due to known postural differences in VO_2_ kinetics [42] and lactate [17], the supine training group trained under higher relative metabolic stress.

We instructed participants to maintain their habitual physical activity levels. However, we did not verify this throughout the intervention. Participants were encouraged to eat before all training sessions, but we did not control for nutritional status or the exact time of they trained. Additionally, several of our cardiovascular outcomes have reduced sample sizes due to data loss, which raises the risk of type II error in our analyses. It may be worthwhile for future work to clarify the steady-state CO responses to supine exercise with a larger sample size. Finally, it is unknown whether our findings would differ following a longer training intervention or would generalize to other supine exercise models and/or cuff-based BFR.

Although we aimed to address the underrepresentation of female participants in exercise research [43], we did not examine sex-specific responses. Our small sample size of males in each training group (n=3) precluded proper assessment of sex-based differences in the current study. For example, mitochondrial O_2_ consumption can be lower in females compared with males at physiological [ADP] [44]. At a given workload, females can have higher [ADP] than males [45]. Although we did not measure [ADP], a potentially higher intracellular [phosphate] in females could have helped explain the increased RPE during supine training via impaired cross-bridge cycling and calcium release [46], 47]. However, this remains an unlikely explanation given that RPE does not differ between females and males during aerobic exercise [48]. Adequately powered comparisons using sex-disaggregated data [49] are needed to help elucidate potential sex-based differences in the mechanisms underpinning adaptations to supine aerobic exercise.

## Conclusions

Our exploratory analyses suggest that training in a supine position with legs elevated above the heart may augment peak work rate in the supine posture without augmenting the mitochondrial proteins measured in this study. Future research should assess indices of mitochondrial remodelling beyond those measured in our study to better assess the effect of supine training on mitochondrial content and function. The absence of observed central cardiovascular adaptations calls for further investigation into the mechanisms by which training in a supine posture with legs above the heart can enhance exercise tolerance.

## Supplementary Material

Supplementary Material Details
