# Supplementary material for: Mitochondrial and cardiovascular responses to aerobic exercise training in supine and upright positions in healthy young adults: a randomized parallel arm trial
Source: Transl Exerc Biomed. 2025 Mar 26;2(1):9–20. doi: 10.1515/teb-2025-0002 (PMC11987498; doi:10.1515/teb-2025-0002)
Supplement: Supplementary file 1 — Supplementary Material Details [file j_teb-2025-0002_suppl_001.pptx]

## Slide 1
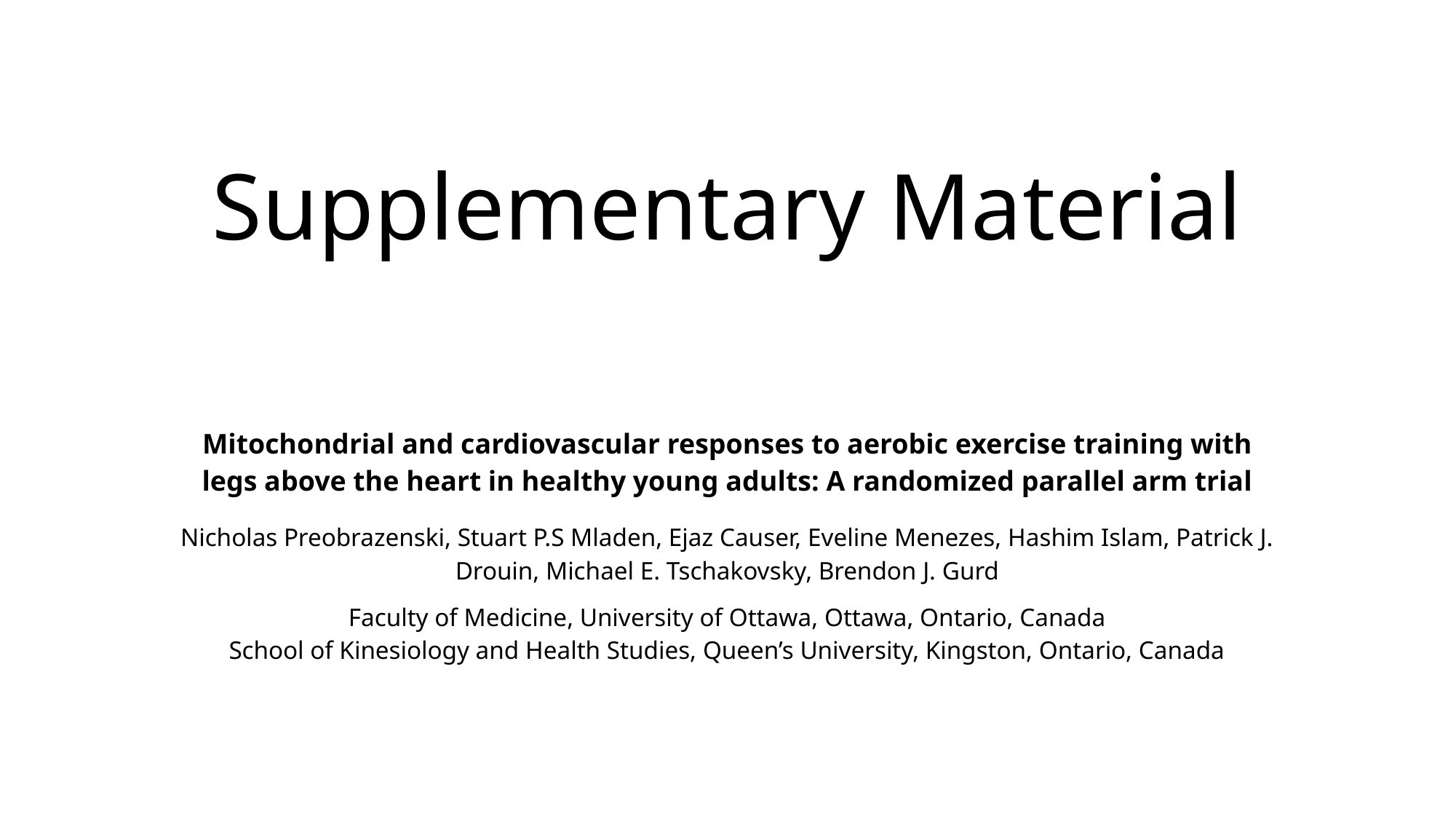

# Supplementary Material
| Mitochondrial and cardiovascular responses to aerobic exercise training with legs above the heart in healthy young adults: A randomized parallel arm trial |
| --- |
| Nicholas Preobrazenski, Stuart P.S Mladen, Ejaz Causer, Eveline Menezes, Hashim Islam, Patrick J. Drouin, Michael E. Tschakovsky, Brendon J. Gurd |
| Faculty of Medicine, University of Ottawa, Ottawa, Ontario, Canada School of Kinesiology and Health Studies, Queen’s University, Kingston, Ontario, Canada |

## Slide 2
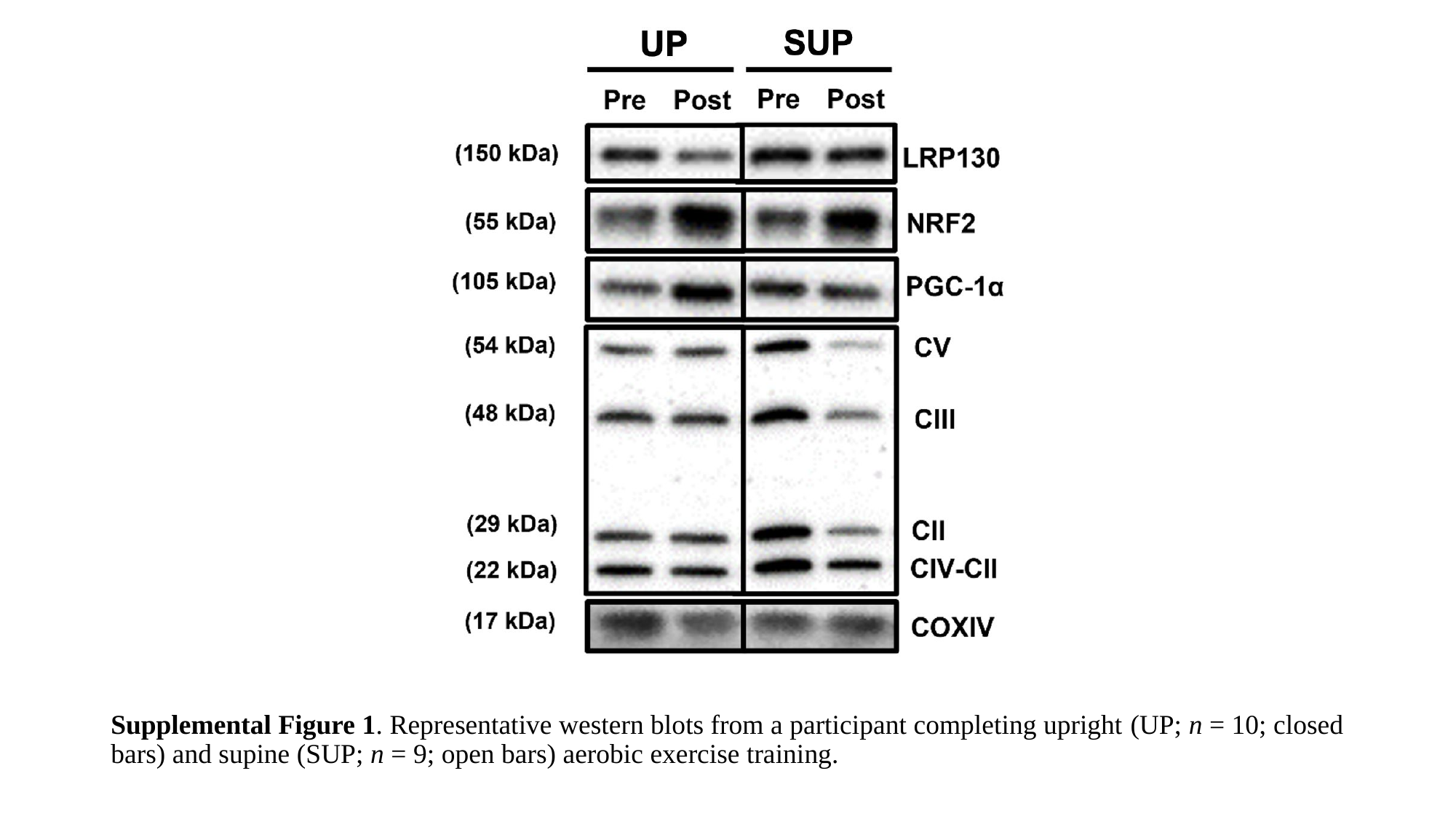

# Supplemental Figure 1. Representative western blots from a participant completing upright (UP; n = 10; closed bars) and supine (SUP; n = 9; open bars) aerobic exercise training.

## Slide 3
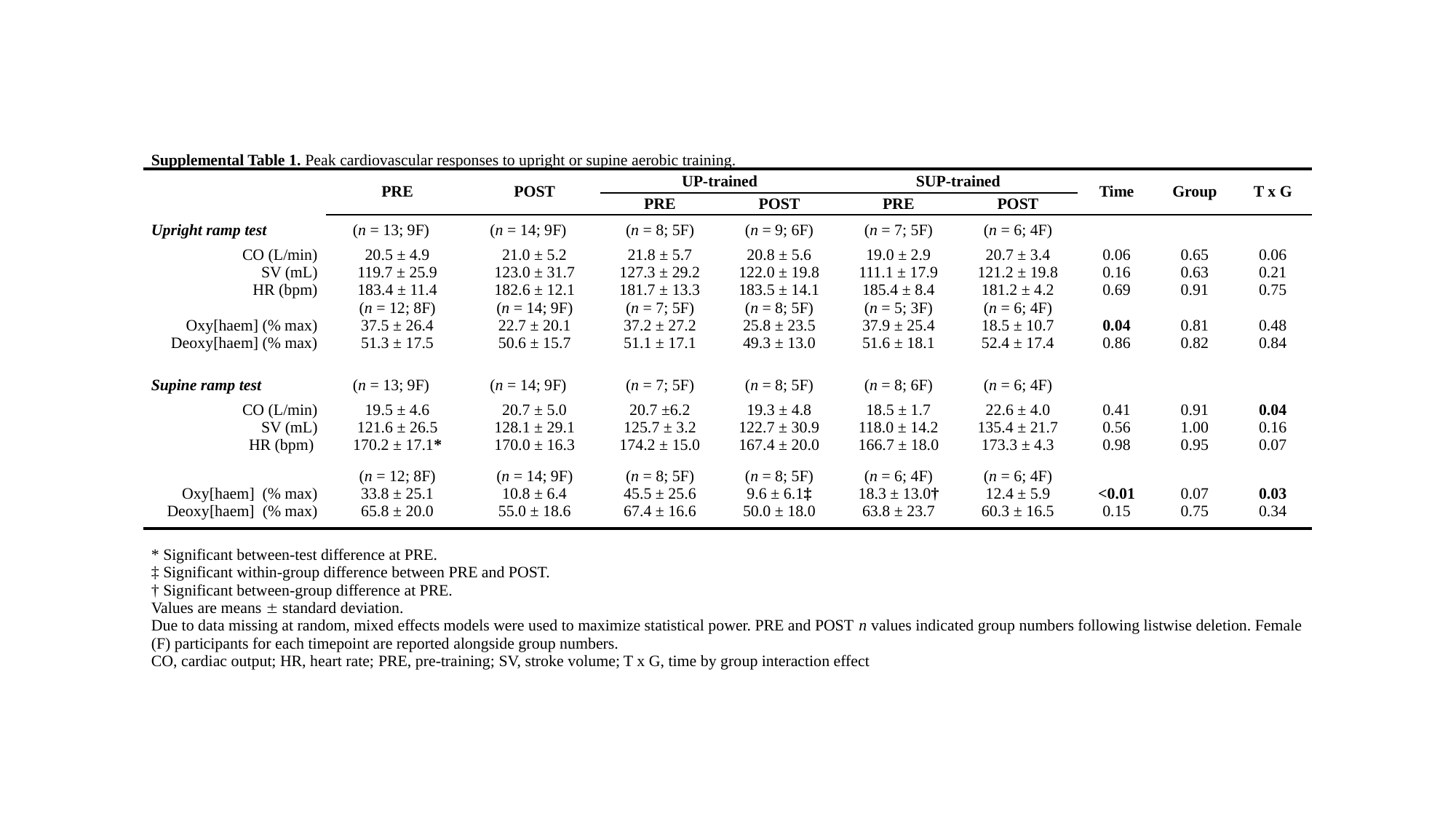

| Supplemental Table 1. Peak cardiovascular responses to upright or supine aerobic training. | | | | | | | | | | |
| --- | --- | --- | --- | --- | --- | --- | --- | --- | --- | --- |
| | PRE | | POST | UP-trained | | SUP-trained | | Time | Group | T x G |
| | | | | PRE | POST | PRE | POST | | | |
| Upright ramp test | (n = 13; 9F) | (n = 14; 9F) | | (n = 8; 5F) | (n = 9; 6F) | (n = 7; 5F) | (n = 6; 4F) | | | |
| CO (L/min) | 20.5 ± 4.9 | | 21.0 ± 5.2 | 21.8 ± 5.7 | 20.8 ± 5.6 | 19.0 ± 2.9 | 20.7 ± 3.4 | 0.06 | 0.65 | 0.06 |
| SV (mL) | 119.7 ± 25.9 | | 123.0 ± 31.7 | 127.3 ± 29.2 | 122.0 ± 19.8 | 111.1 ± 17.9 | 121.2 ± 19.8 | 0.16 | 0.63 | 0.21 |
| HR (bpm) | 183.4 ± 11.4 | | 182.6 ± 12.1 | 181.7 ± 13.3 | 183.5 ± 14.1 | 185.4 ± 8.4 | 181.2 ± 4.2 | 0.69 | 0.91 | 0.75 |
| | (n = 12; 8F) | | (n = 14; 9F) | (n = 7; 5F) | (n = 8; 5F) | (n = 5; 3F) | (n = 6; 4F) | | | |
| Oxy[haem] (% max) | 37.5 ± 26.4 | | 22.7 ± 20.1 | 37.2 ± 27.2 | 25.8 ± 23.5 | 37.9 ± 25.4 | 18.5 ± 10.7 | 0.04 | 0.81 | 0.48 |
| Deoxy[haem] (% max) | 51.3 ± 17.5 | | 50.6 ± 15.7 | 51.1 ± 17.1 | 49.3 ± 13.0 | 51.6 ± 18.1 | 52.4 ± 17.4 | 0.86 | 0.82 | 0.84 |
| | | | | | | | | | | |
| Supine ramp test | (n = 13; 9F) | (n = 14; 9F) | | (n = 7; 5F) | (n = 8; 5F) | (n = 8; 6F) | (n = 6; 4F) | | | |
| CO (L/min) | 19.5 ± 4.6 | | 20.7 ± 5.0 | 20.7 ±6.2 | 19.3 ± 4.8 | 18.5 ± 1.7 | 22.6 ± 4.0 | 0.41 | 0.91 | 0.04 |
| SV (mL) | 121.6 ± 26.5 | | 128.1 ± 29.1 | 125.7 ± 3.2 | 122.7 ± 30.9 | 118.0 ± 14.2 | 135.4 ± 21.7 | 0.56 | 1.00 | 0.16 |
| HR (bpm) | 170.2 ± 17.1\* | | 170.0 ± 16.3 | 174.2 ± 15.0 | 167.4 ± 20.0 | 166.7 ± 18.0 | 173.3 ± 4.3 | 0.98 | 0.95 | 0.07 |
| | (n = 12; 8F) | | (n = 14; 9F) | (n = 8; 5F) | (n = 8; 5F) | (n = 6; 4F) | (n = 6; 4F) | | | |
| Oxy[haem] (% max) | 33.8 ± 25.1 | | 10.8 ± 6.4 | 45.5 ± 25.6 | 9.6 ± 6.1‡ | 18.3 ± 13.0† | 12.4 ± 5.9 | <0.01 | 0.07 | 0.03 |
| Deoxy[haem] (% max) | 65.8 ± 20.0 | | 55.0 ± 18.6 | 67.4 ± 16.6 | 50.0 ± 18.0 | 63.8 ± 23.7 | 60.3 ± 16.5 | 0.15 | 0.75 | 0.34 |
| \* Significant between-test difference at PRE. ‡ Significant within-group difference between PRE and POST. † Significant between-group difference at PRE. Values are means ± standard deviation. Due to data missing at random, mixed effects models were used to maximize statistical power. PRE and POST n values indicated group numbers following listwise deletion. Female (F) participants for each timepoint are reported alongside group numbers. CO, cardiac output; HR, heart rate; PRE, pre-training; SV, stroke volume; T x G, time by group interaction effect | | | | | | | | | | |

## Slide 4
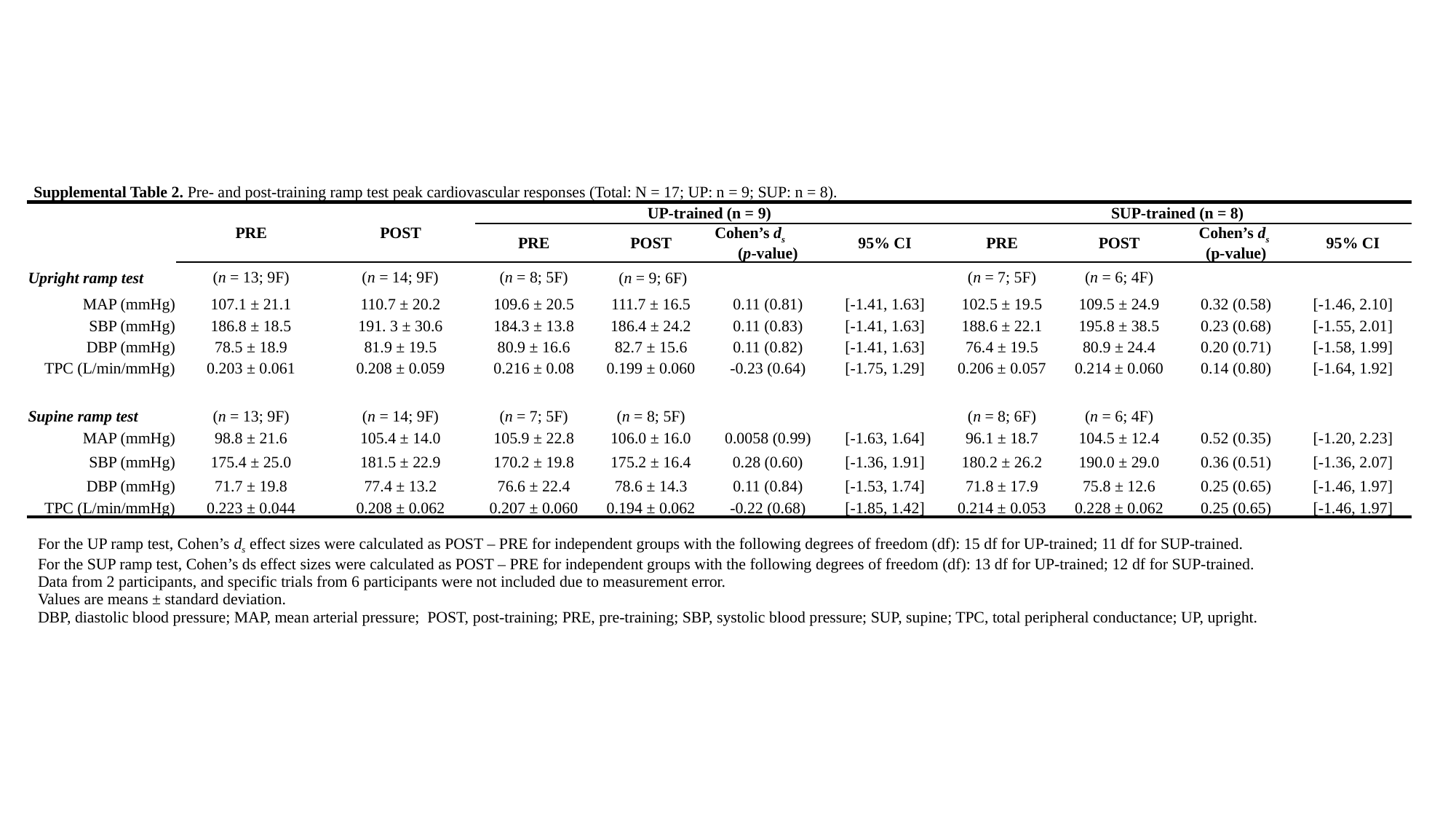

| Supplemental Table 2. Pre- and post-training ramp test peak cardiovascular responses (Total: N = 17; UP: n = 9; SUP: n = 8). | | | | | | | | | | | |
| --- | --- | --- | --- | --- | --- | --- | --- | --- | --- | --- | --- |
| | PRE | POST | | UP-trained (n = 9) | | | | SUP-trained (n = 8) | | | |
| | | | | PRE | POST | Cohen’s ds (p-value) | 95% CI | PRE | POST | Cohen’s ds (p-value) | 95% CI |
| Upright ramp test | (n = 13; 9F) | (n = 14; 9F) | (n = 8; 5F) | | (n = 9; 6F) | | | (n = 7; 5F) | (n = 6; 4F) | | |
| MAP (mmHg) | 107.1 ± 21.1 | 110.7 ± 20.2 | | 109.6 ± 20.5 | 111.7 ± 16.5 | 0.11 (0.81) | [-1.41, 1.63] | 102.5 ± 19.5 | 109.5 ± 24.9 | 0.32 (0.58) | [-1.46, 2.10] |
| SBP (mmHg) | 186.8 ± 18.5 | 191. 3 ± 30.6 | | 184.3 ± 13.8 | 186.4 ± 24.2 | 0.11 (0.83) | [-1.41, 1.63] | 188.6 ± 22.1 | 195.8 ± 38.5 | 0.23 (0.68) | [-1.55, 2.01] |
| DBP (mmHg) | 78.5 ± 18.9 | 81.9 ± 19.5 | | 80.9 ± 16.6 | 82.7 ± 15.6 | 0.11 (0.82) | [-1.41, 1.63] | 76.4 ± 19.5 | 80.9 ± 24.4 | 0.20 (0.71) | [-1.58, 1.99] |
| TPC (L/min/mmHg) | 0.203 ± 0.061 | 0.208 ± 0.059 | | 0.216 ± 0.08 | 0.199 ± 0.060 | -0.23 (0.64) | [-1.75, 1.29] | 0.206 ± 0.057 | 0.214 ± 0.060 | 0.14 (0.80) | [-1.64, 1.92] |
| Supine ramp test | (n = 13; 9F) | (n = 14; 9F) | | (n = 7; 5F) | (n = 8; 5F) | | | (n = 8; 6F) | (n = 6; 4F) | | |
| MAP (mmHg) | 98.8 ± 21.6 | 105.4 ± 14.0 | | 105.9 ± 22.8 | 106.0 ± 16.0 | 0.0058 (0.99) | [-1.63, 1.64] | 96.1 ± 18.7 | 104.5 ± 12.4 | 0.52 (0.35) | [-1.20, 2.23] |
| SBP (mmHg) | 175.4 ± 25.0 | 181.5 ± 22.9 | | 170.2 ± 19.8 | 175.2 ± 16.4 | 0.28 (0.60) | [-1.36, 1.91] | 180.2 ± 26.2 | 190.0 ± 29.0 | 0.36 (0.51) | [-1.36, 2.07] |
| DBP (mmHg) | 71.7 ± 19.8 | 77.4 ± 13.2 | | 76.6 ± 22.4 | 78.6 ± 14.3 | 0.11 (0.84) | [-1.53, 1.74] | 71.8 ± 17.9 | 75.8 ± 12.6 | 0.25 (0.65) | [-1.46, 1.97] |
| TPC (L/min/mmHg) | 0.223 ± 0.044 | 0.208 ± 0.062 | | 0.207 ± 0.060 | 0.194 ± 0.062 | -0.22 (0.68) | [-1.85, 1.42] | 0.214 ± 0.053 | 0.228 ± 0.062 | 0.25 (0.65) | [-1.46, 1.97] |
| For the UP ramp test, Cohen’s ds effect sizes were calculated as POST – PRE for independent groups with the following degrees of freedom (df): 15 df for UP-trained; 11 df for SUP-trained. For the SUP ramp test, Cohen’s ds effect sizes were calculated as POST – PRE for independent groups with the following degrees of freedom (df): 13 df for UP-trained; 12 df for SUP-trained. Data from 2 participants, and specific trials from 6 participants were not included due to measurement error. Values are means ± standard deviation. DBP, diastolic blood pressure; MAP, mean arterial pressure; POST, post-training; PRE, pre-training; SBP, systolic blood pressure; SUP, supine; TPC, total peripheral conductance; UP, upright. | | | | | | | | | | | |

## Slide 5
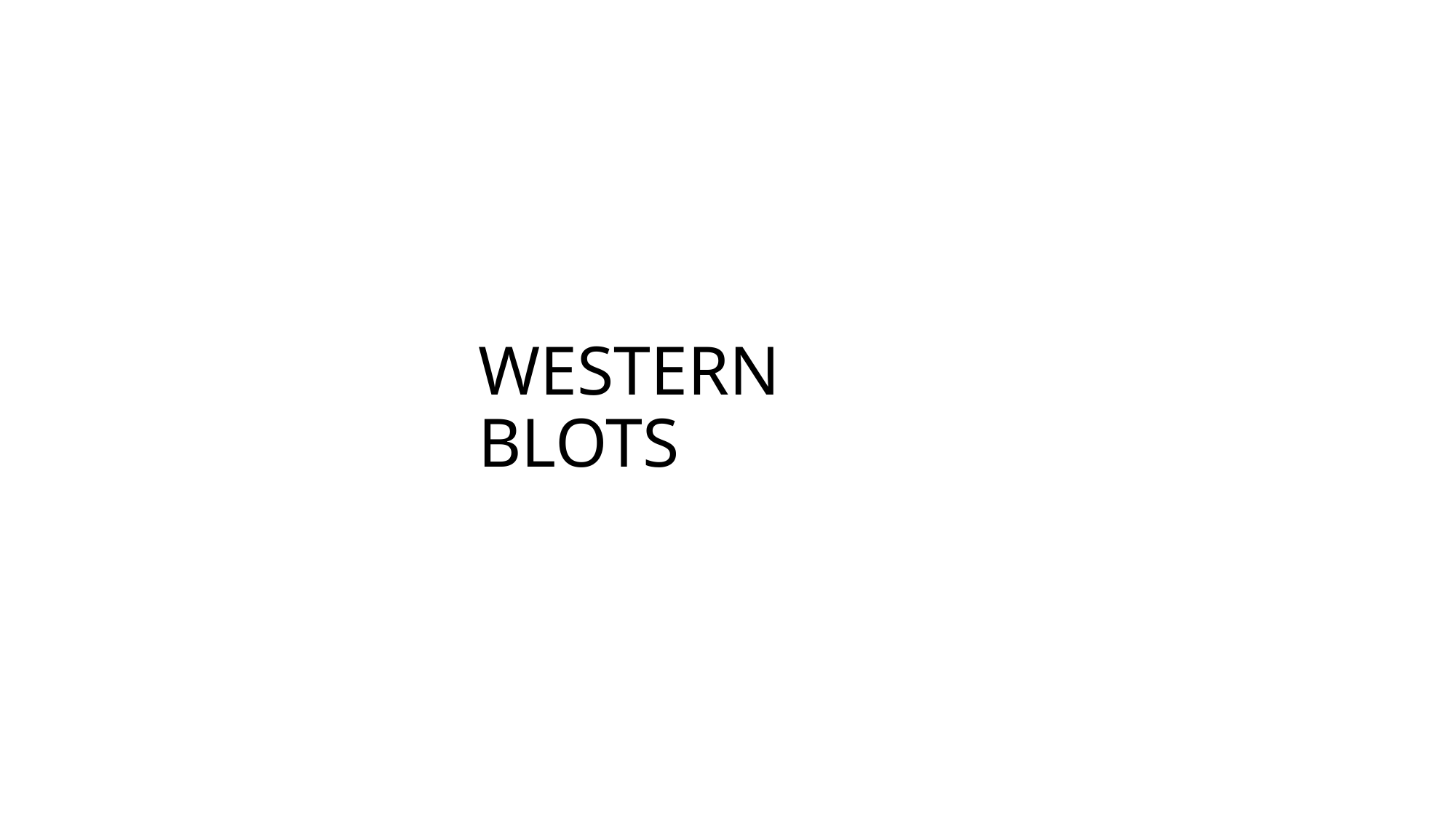

# WESTERN BLOTS

## Slide 6
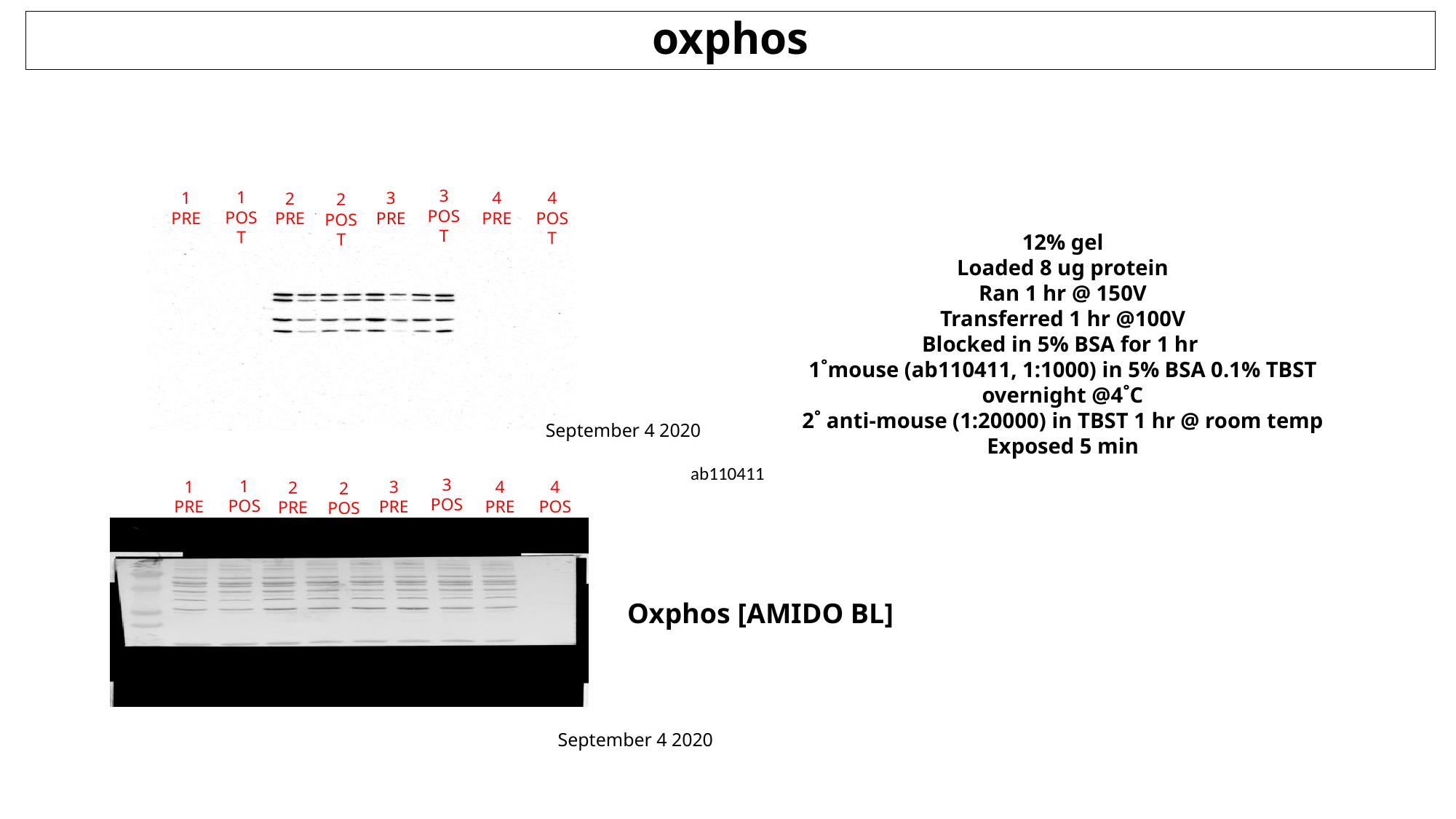

# oxphos
3 POST
1 POST
1 PRE
3 PRE
4 POST
4 PRE
2 PRE
2 POST
12% gel
Loaded 8 ug protein
Ran 1 hr @ 150V
Transferred 1 hr @100V
Blocked in 5% BSA for 1 hr
1˚mouse (ab110411, 1:1000) in 5% BSA 0.1% TBST overnight @4˚C
2˚ anti-mouse (1:20000) in TBST 1 hr @ room temp
Exposed 5 min
September 4 2020
| ab110411 |
| --- |
3 POST
1 POST
1 PRE
3 PRE
4 POST
4 PRE
2 PRE
2 POST
Oxphos [AMIDO BL]
September 4 2020

## Slide 7
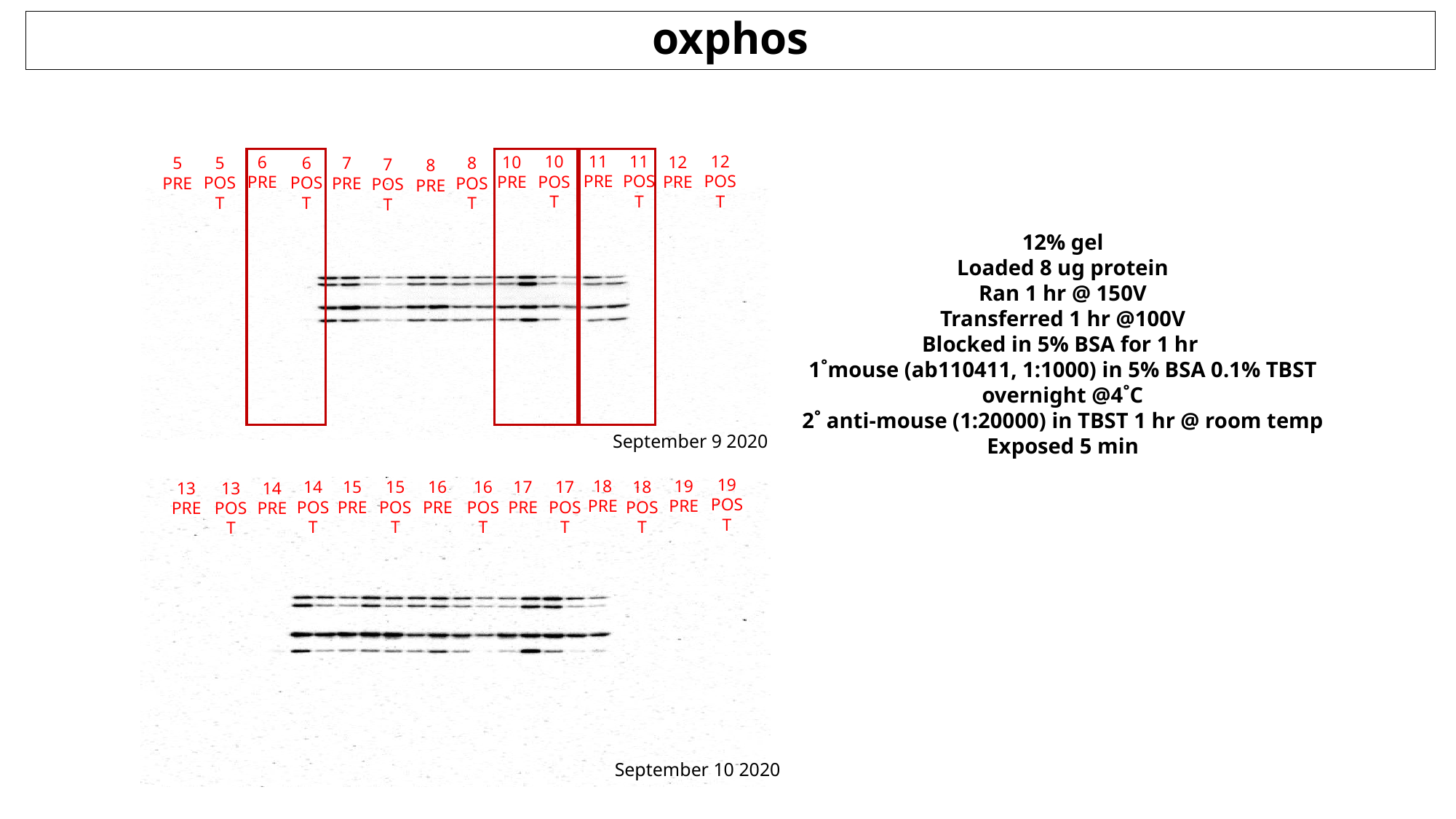

# oxphos
11 POST
12 POST
11 PRE
10 POST
12 PRE
6 PRE
10 PRE
6 POST
5 POST
8 POST
5 PRE
7 PRE
7 POST
8 PRE
12% gel
Loaded 8 ug protein
Ran 1 hr @ 150V
Transferred 1 hr @100V
Blocked in 5% BSA for 1 hr
1˚mouse (ab110411, 1:1000) in 5% BSA 0.1% TBST overnight @4˚C
2˚ anti-mouse (1:20000) in TBST 1 hr @ room temp
Exposed 5 min
September 9 2020
19 POST
19 PRE
18 PRE
18 POST
17 POST
17 PRE
16 POST
16 PRE
15 POST
15 PRE
14 POST
13 PRE
14 PRE
13 POST
September 10 2020

## Slide 8
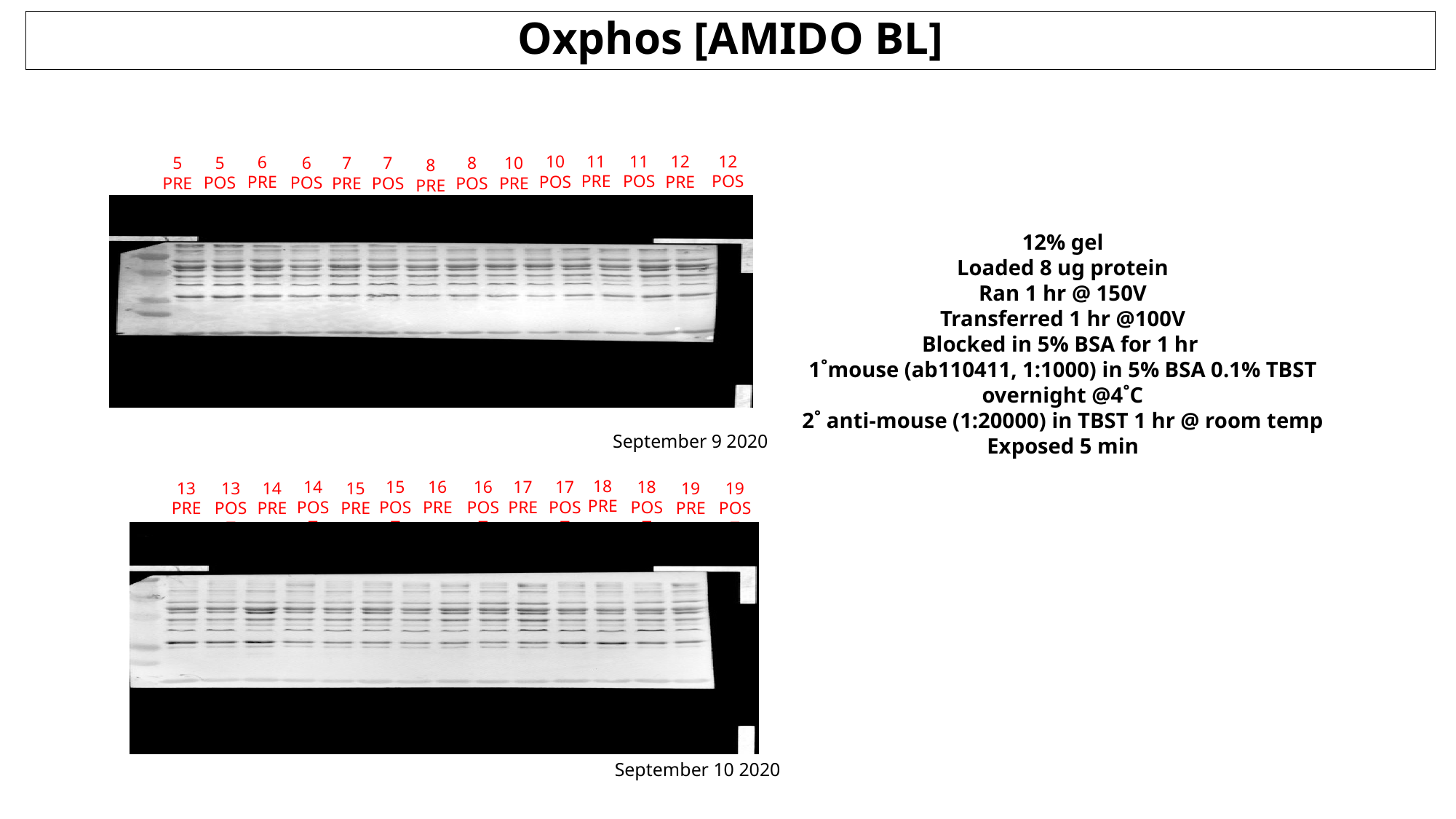

# Oxphos [AMIDO BL]
11 POST
11 PRE
12 POST
10 POST
12 PRE
6 PRE
6 POST
5 POST
7 POST
8 POST
10 PRE
5 PRE
7 PRE
8 PRE
12% gel
Loaded 8 ug protein
Ran 1 hr @ 150V
Transferred 1 hr @100V
Blocked in 5% BSA for 1 hr
1˚mouse (ab110411, 1:1000) in 5% BSA 0.1% TBST overnight @4˚C
2˚ anti-mouse (1:20000) in TBST 1 hr @ room temp
Exposed 5 min
September 9 2020
18 PRE
18 POST
17 POST
17 PRE
16 POST
16 PRE
15 POST
14 POST
13 PRE
15 PRE
19 PRE
19 POST
14 PRE
13 POST
September 10 2020

## Slide 9
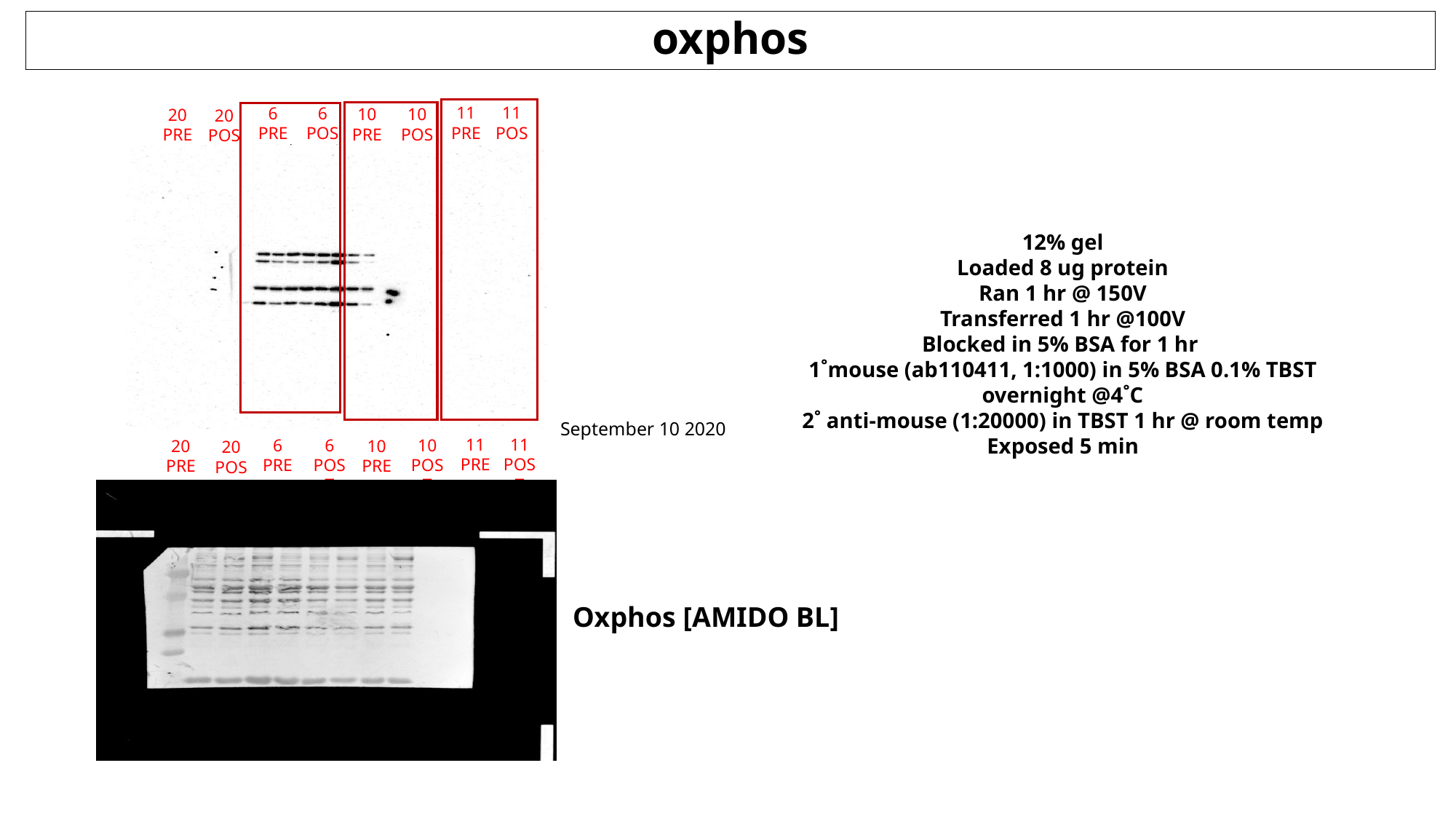

# oxphos
11 POST
11 PRE
6 POST
6 PRE
10 PRE
10 POST
20 PRE
20 POST
12% gel
Loaded 8 ug protein
Ran 1 hr @ 150V
Transferred 1 hr @100V
Blocked in 5% BSA for 1 hr
1˚mouse (ab110411, 1:1000) in 5% BSA 0.1% TBST overnight @4˚C
2˚ anti-mouse (1:20000) in TBST 1 hr @ room temp
Exposed 5 min
September 10 2020
11 PRE
11 POST
10 POST
6 POST
6 PRE
20 PRE
10 PRE
20 POST
Oxphos [AMIDO BL]

## Slide 10
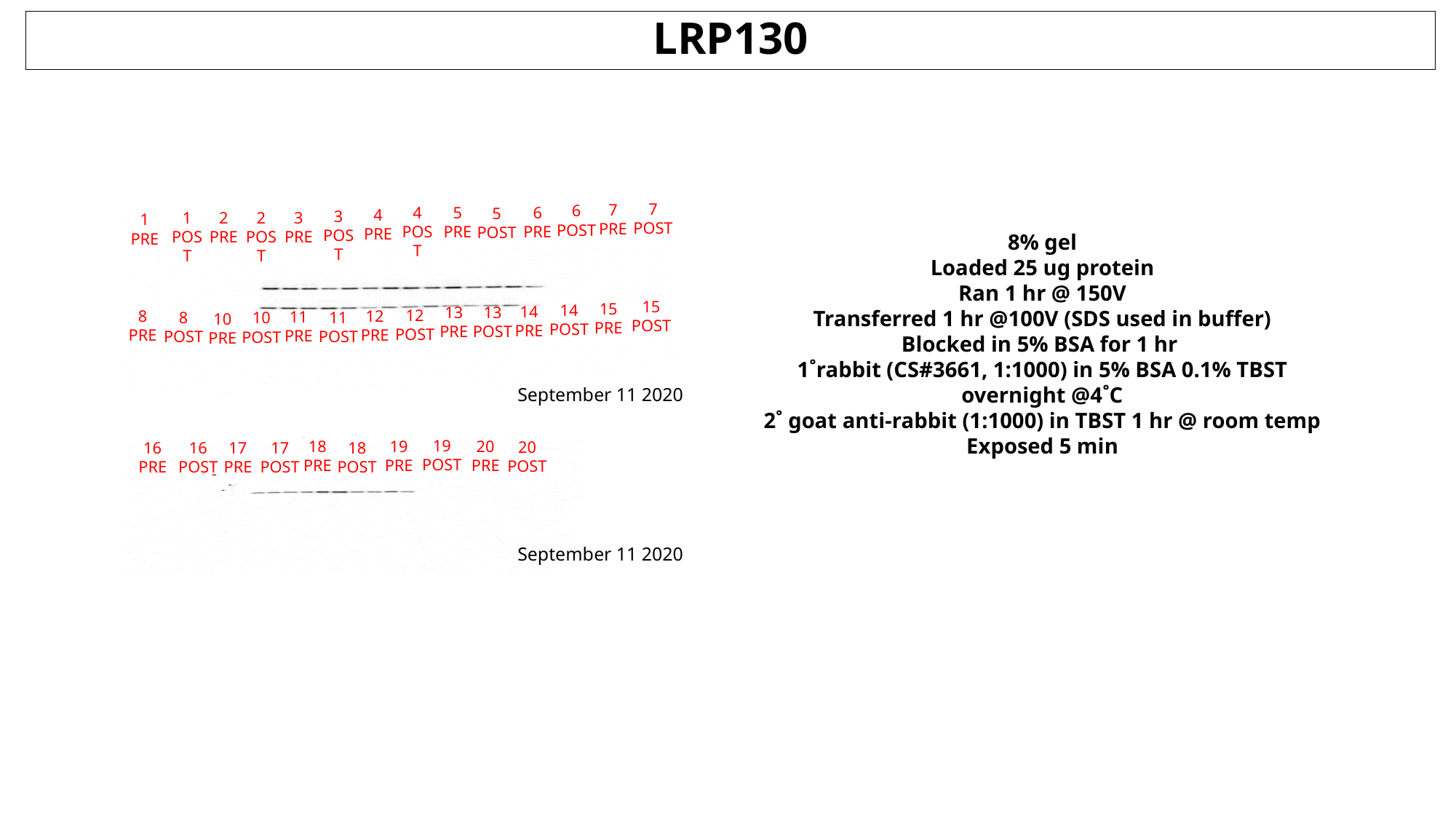

# LRP130
7 POST
7 PRE
6 POST
6 PRE
5 PRE
4 POST
5 POST
4 PRE
3 POST
2 POST
3 PRE
1 POST
2 PRE
1 PRE
8% gel
Loaded 25 ug protein
Ran 1 hr @ 150V
Transferred 1 hr @100V (SDS used in buffer)
Blocked in 5% BSA for 1 hr
1˚rabbit (CS#3661, 1:1000) in 5% BSA 0.1% TBST overnight @4˚C
2˚ goat anti-rabbit (1:1000) in TBST 1 hr @ room temp
Exposed 5 min
15 POST
15 PRE
14 POST
14 PRE
13 POST
13 PRE
12 POST
12 PRE
8 PRE
11 PRE
8 POST
11 POST
10 POST
10 PRE
September 11 2020
19 POST
19 PRE
20 PRE
18 PRE
20 POST
18 POST
17 POST
17 PRE
16 POST
16 PRE
September 11 2020

## Slide 11
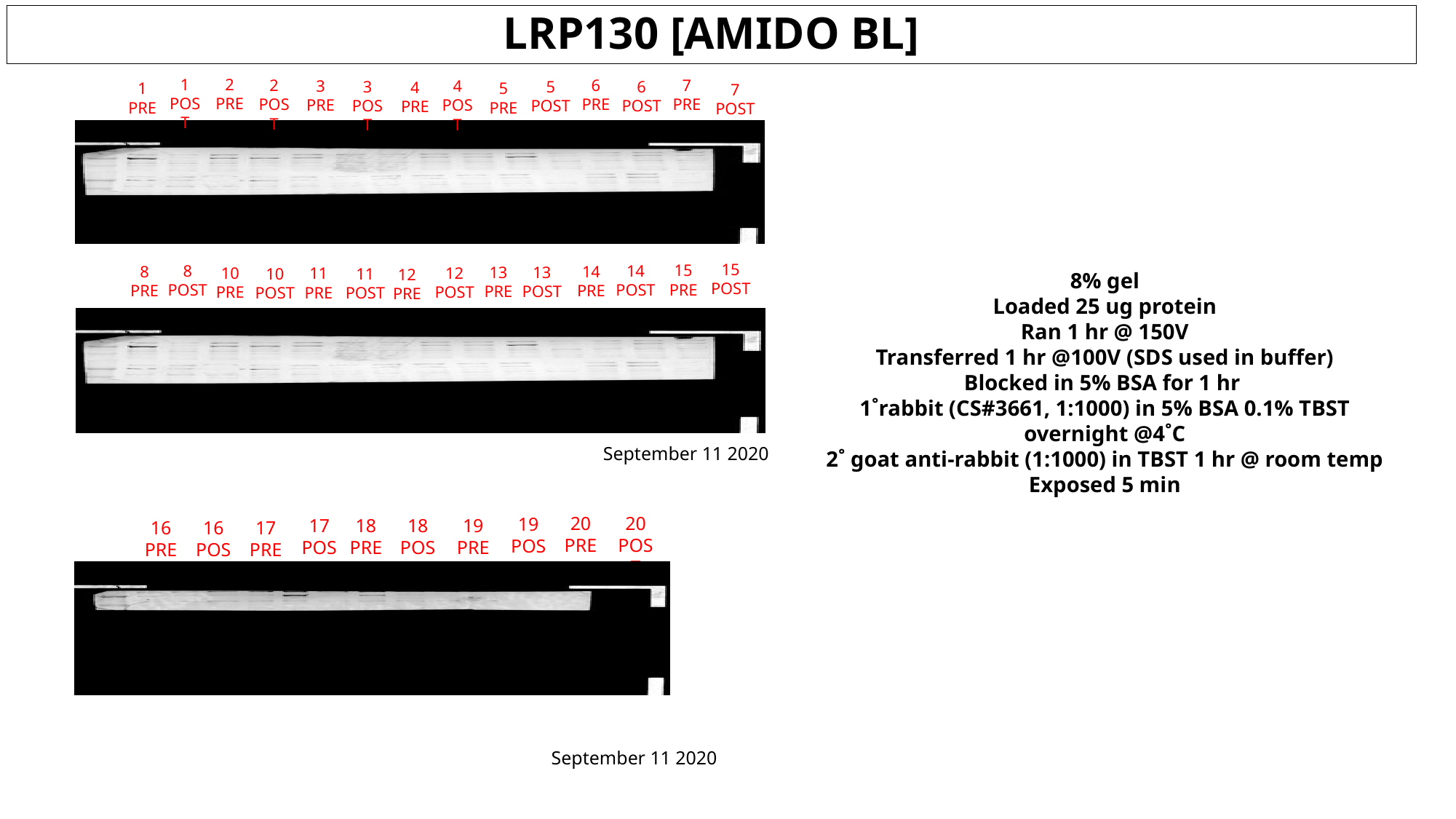

# LRP130 [AMIDO BL]
1 POST
2 PRE
2 POST
6 PRE
7 PRE
3 PRE
4 POST
3 POST
5 POST
6 POST
4 PRE
1 PRE
5 PRE
7 POST
15 POST
15 PRE
14 POST
8 POST
8 PRE
14 PRE
13 POST
13 PRE
12 POST
10 PRE
11 PRE
10 POST
11 POST
12 PRE
8% gel
Loaded 25 ug protein
Ran 1 hr @ 150V
Transferred 1 hr @100V (SDS used in buffer)
Blocked in 5% BSA for 1 hr
1˚rabbit (CS#3661, 1:1000) in 5% BSA 0.1% TBST overnight @4˚C
2˚ goat anti-rabbit (1:1000) in TBST 1 hr @ room temp
Exposed 5 min
September 11 2020
20 POST
20 PRE
19 POST
17 POST
18 PRE
18 POST
19 PRE
16 POST
17 PRE
16 PRE
September 11 2020

## Slide 12
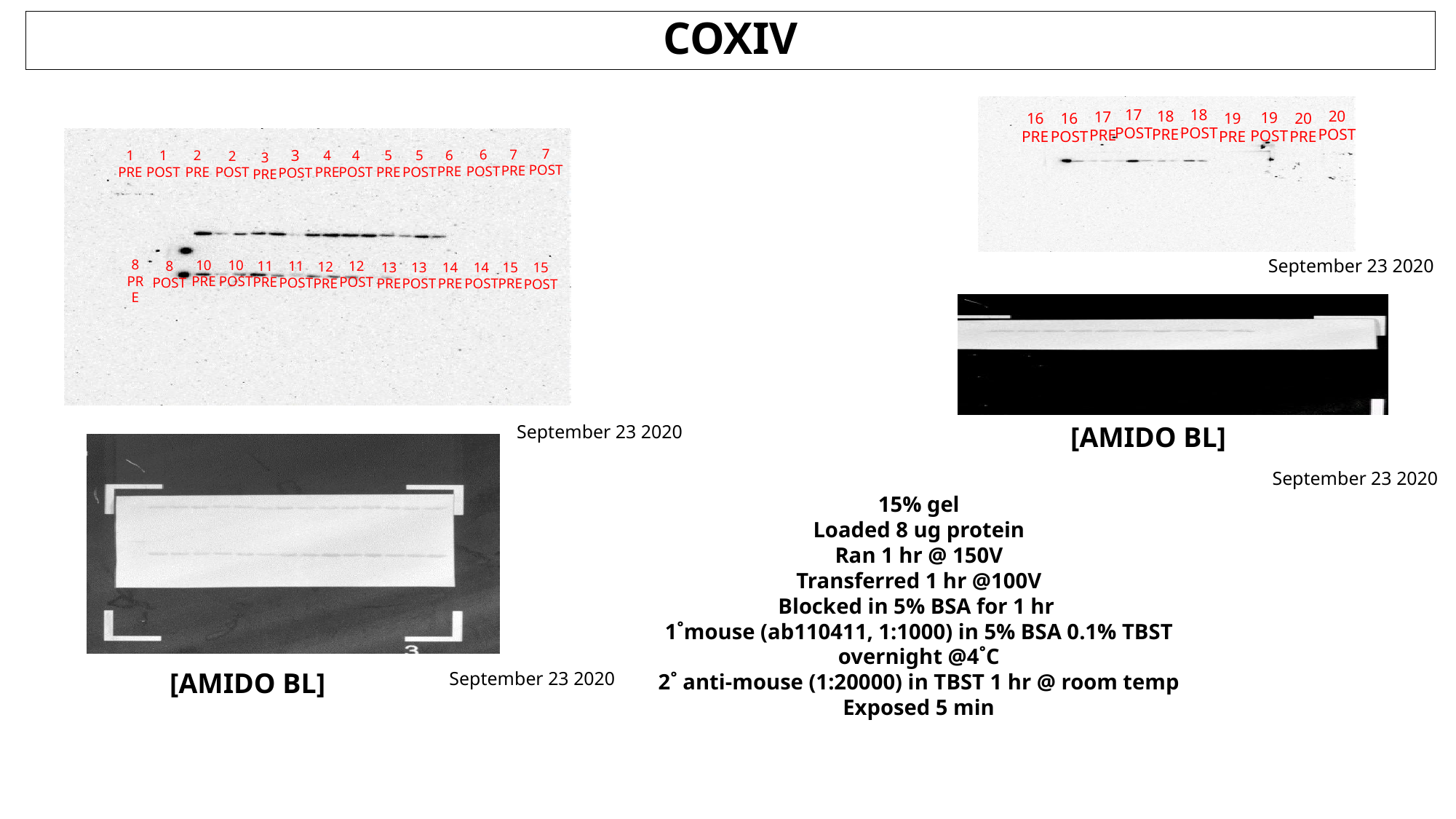

COXIV
17 POST
18 POST
18 PRE
20 POST
17 PRE
19 POST
16 PRE
16 POST
19 PRE
20 PRE
7 POST
7 PRE
3 POST
6 POST
6 PRE
1 PRE
1 POST
2 PRE
4 PRE
4 POST
5 PRE
5 POST
2 POST
3 PRE
September 23 2020
8 PRE
10 POST
10 PRE
11 PRE
12 POST
8 POST
11 POST
12 PRE
13 PRE
13 POST
14 PRE
14 POST
15 PRE
15 POST
September 23 2020
 [AMIDO BL]
September 23 2020
15% gel
Loaded 8 ug protein
Ran 1 hr @ 150V
Transferred 1 hr @100V
Blocked in 5% BSA for 1 hr
1˚mouse (ab110411, 1:1000) in 5% BSA 0.1% TBST overnight @4˚C
2˚ anti-mouse (1:20000) in TBST 1 hr @ room temp
Exposed 5 min
 [AMIDO BL]
September 23 2020

## Slide 13
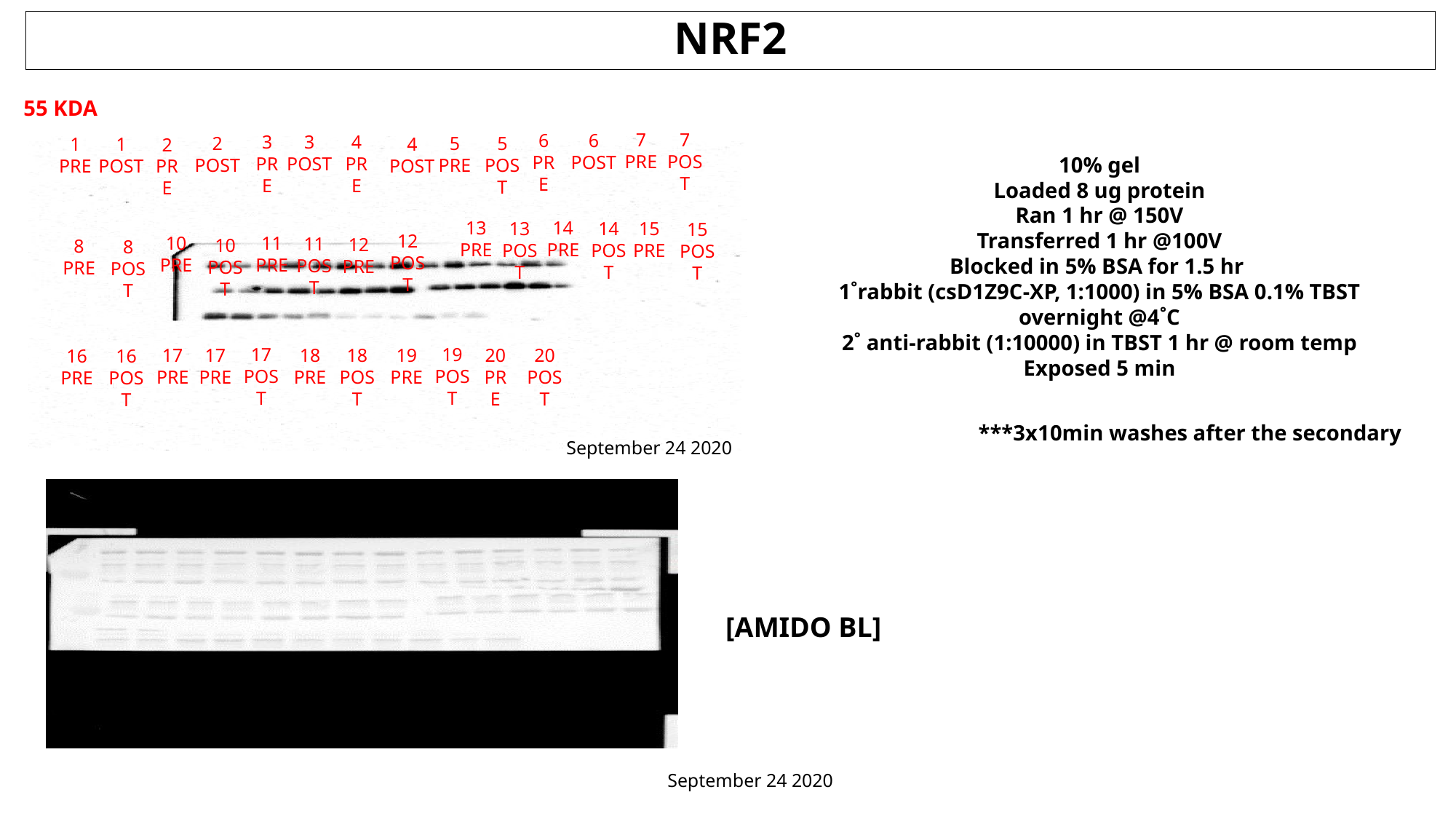

NRF2
55 KDA
7 POST
7 PRE
6 PRE
6 POST
4 PRE
3 POST
3 PRE
5 POST
2 POST
5 PRE
1 POST
1 PRE
4 POST
2 PRE
10% gel
Loaded 8 ug protein
Ran 1 hr @ 150V
Transferred 1 hr @100V
Blocked in 5% BSA for 1.5 hr
1˚rabbit (csD1Z9C-XP, 1:1000) in 5% BSA 0.1% TBST overnight @4˚C
2˚ anti-rabbit (1:10000) in TBST 1 hr @ room temp
Exposed 5 min
13 PRE
14 PRE
14 POST
13 POST
15 PRE
15 POST
12 POST
10 PRE
11 PRE
11 POST
12 PRE
10 POST
8 PRE
8 POST
17 POST
19 POST
17 PRE
17 PRE
18 PRE
18 POST
19 PRE
20 PRE
20 POST
16 PRE
16 POST
***3x10min washes after the secondary
September 24 2020
 [AMIDO BL]
September 24 2020

## Slide 14
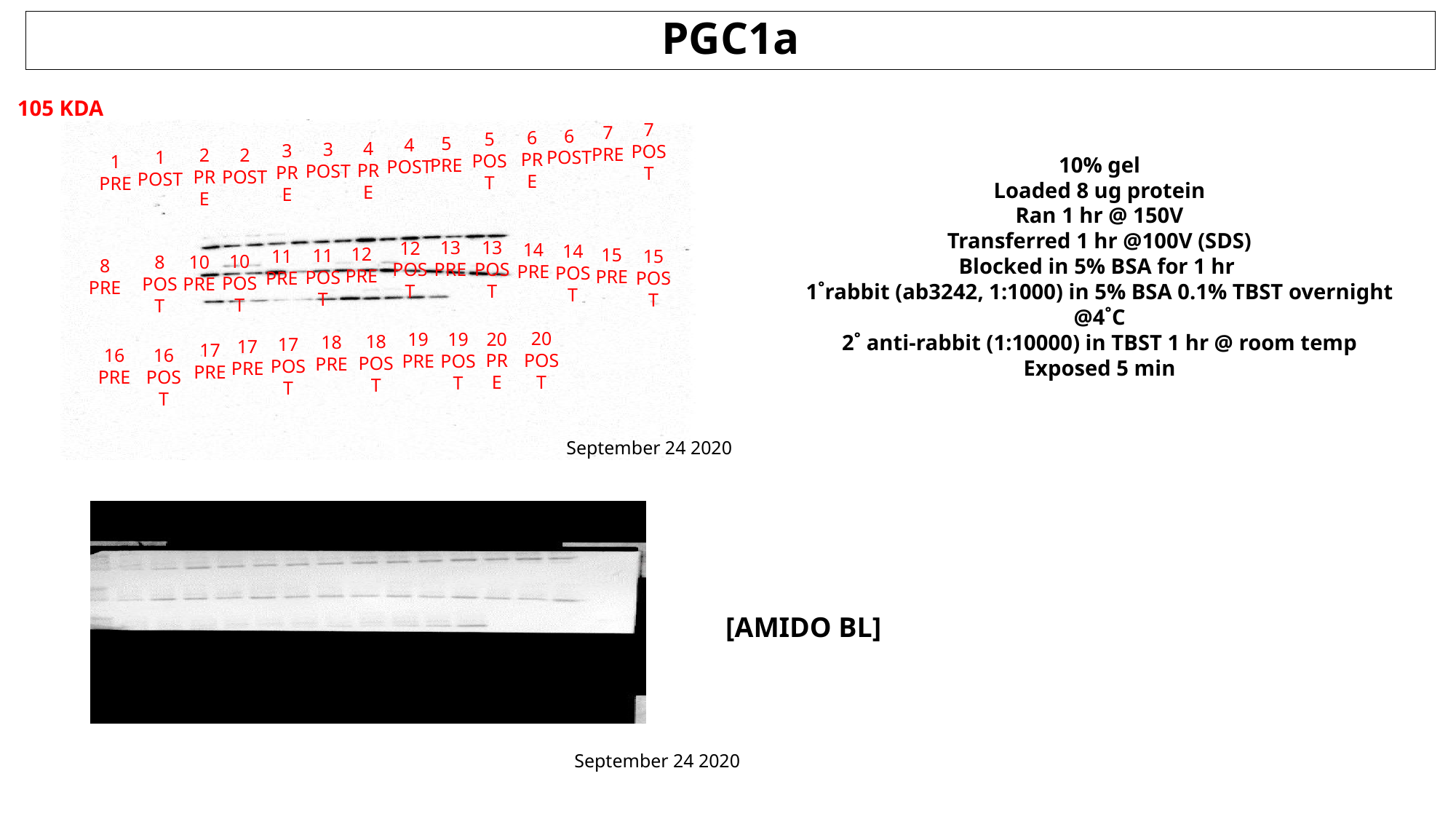

PGC1a
105 KDA
7 POST
7 PRE
6 POST
6 PRE
5 POST
5 PRE
4 POST
4 PRE
3 POST
3 PRE
2 PRE
2 POST
1 POST
1 PRE
10% gel
Loaded 8 ug protein
Ran 1 hr @ 150V
Transferred 1 hr @100V (SDS)
Blocked in 5% BSA for 1 hr
1˚rabbit (ab3242, 1:1000) in 5% BSA 0.1% TBST overnight @4˚C
2˚ anti-rabbit (1:10000) in TBST 1 hr @ room temp
Exposed 5 min
13 POST
12 POST
13 PRE
14 PRE
14 POST
12 PRE
15 PRE
11 POST
11 PRE
15 POST
10 POST
10 PRE
8 POST
8 PRE
20 POST
20 PRE
19 POST
19 PRE
18 POST
18 PRE
17 POST
17 PRE
17 PRE
16 POST
16 PRE
September 24 2020
 [AMIDO BL]
September 24 2020
